# Genomic epidemiology and immune escape of SARS-CoV-2 recombinant strains circulating in Botswana

**DOI:** 10.1016/j.ijregi.2024.100484

**Published:** 2024-11-02

**Authors:** Wonderful T. Choga, Irene Gobe, Kedumetse Seru, Dorcas Maruapula, Nokuthula S. Ndlovu, Boitumelo J. L Zuze, Patience Motshosi, Teko Matsuru, Phenyo Sabone, Xiaoyu Yu, Jason T. Blackard, James E. San, Joseph Makhema, Simani Gaseitsiwe, Sikhulile Moyo

**Affiliations:** 1 Botswana Harvard Health Partnership, Gaborone, Botswana; 2 School of Allied Health Sciences, Faculty of Health Sciences, Gaborone, Botswana; 3 Institute of Evolutionary Biology, University of Edinburgh, Edinburgh, Scotland, UK; 4 University of Cincinnati College of Medicine, Cincinnati, USA; 5 Duke Human Vaccine Institute (DHVI), Durham, USA; 6 University of KwaZulu-Natal (UKZN), Durban, South Africa; 7 Department of Immunology and Infectious Diseases, Harvard T.H. Chan School of Public Health, Boston, USA; 8 School of Health Systems and Public Health, University of Pretoria, Pretoria, South Africa; 9 Division of Medical Virology, Faculty of Medicine and Health Sciences, Stellenbosch University, Tygerberg, South Africa

**Keywords:** COVID-19, SARS-CoV-2, Recombination, Immune escape, Botswana, Africa

## Abstract

**Objectives:**

We characterized the molecular and mutational landscape of SARS-CoV-2 recombinant strains in Botswana.

**Methods:**

We performed genomic, phylogenetic, and immunoinformatic analyses of 5254 near-complete genomes from 2020 to 2023. We assessed the presence of mutations of interested (MutOI) that may be associated with immune escape *in silico*.

**Results:**

We observed a few recombinant strains in Botswana, with the majority being descendants of Omicron (XBB*), except for XV and XM. Most recombinant sequences corresponded to transmission clusters. Most recombination events occurred within the receptor-binding domain (RDB) of the spike (S) protein. We identified 16 MutOI among different proteins, with the majority occurring at a very low global prevalence (<4.8 × 10^−^⁵). We also observed S:Q474K, a MutOI in the RBD, that was predicted to escape human leukocyte antigen class I–mediated immune responses. Molecular surveillance is vital to inform early detection and response to potential variants with heightened immune and vaccine breakthrough properties.

**Conclusions:**

These results underscore the need for continued molecular surveillance to map the evolutionary landscape of SARS-CoV-2.

## Highlights

• There is a low prevalence of circulating SARS-CoV-2 recombinant sequences in Botswana.
• XBB* cases were the most predominant SARS-CoV-2 recombinants cases.
• We report the first cases of XBJ.1.1 and XM recombinant sequences in Africa.
• Most recombinants had breaking points in the receptor-binding domain in spike.
• We identified rare mutations of interest, including S:Q474K, that may cause immune escape.

## Introduction

SARS-CoV-2, the causative agent of COVID-19, has continuously evolved into multiple lineages due to viral RNA editing, drug interactions, replication errors, and recombination events [1]. Recombination contributes to the adaptive evolution of the virus and can occur when distinct strains co-circulate in a population, co-infection occurs, and viral genes are reshuffled, giving rise to chimeric strains with altered phenotypes [2]. The emergence of recombinant viruses poses a threat to public health; for instance, recombination can integrate variant-specific features that may lead to treatment resistance, immune escape, and/or increase transmissibility [3]. Emerging SARS-CoV-2 recombination variants were predicted, with the first few cases reported during the early part of the pandemic, including XA—a combination of Alpha and Delta—that was reported in early 2021 in the United Kingdom and Japan [4].

During the early part of the Omicron-driven epidemic wave (November 2021 to January 2022), co-circulation of Delta and Omicron lineages (BA.1 and BA.2) occurred and led to the emergence of inter-variant of concern (VOC) recombinants from a combination of Delta (AY.4) and Omicron (BA.1), e.g. XD and XF [5]. Additional inter-lineage recombination variants also emerged in India in August 2022, including XBB—a combination of BA.2.10.1 (BJ.1) and BM.1.1—and XBB.1—a combination of BJ.1 and BA.2.75 [6]. The descendants of XBB (e.g. XBB.1.16, XBB.1.5) were assigned as variants of interest (VOIs) by the World Health Organization (WHO) in early 2023 [7]. Emerging VOIs could become VOCs and, thus, require further investigation.

Major histocompatibility complexes (MHCs)—known as human leukocyte antigens (HLAs) in humans—are integral components of the host immune system, with HLA class I alleles presenting processed antigenic peptides (epitopes) to clusters of differentiation (CD8^+^) cytolytic T cells (CTLs) and HLA class II presenting to T_h_ cells [8]. Virus-specific T-cell immunity influences the outcome of SARS-CoV-2 infection and emerging evidence suggests that HLA class I–restricted T cells contribute to the control of SARS-CoV-2 and the immunity provided by currently approved vaccines [9]. However, mutations within immunogenic epitopes can affect presentation by HLAs and lead to immune escape. *In silico* analysis provides an alternative, inexpensive, and effective method to characterize HLA epitope complexes when peptides of different amino acid compositions are introduced [10].

Botswana is a middle-income country in southern Africa and has a population of about 2.3 million. Its initial case of SARS-CoV-2 was reported on March 20, 2020. By June 20, 2024, the country had reported 330,683 cases and 2,801 fatalities. Botswana has achieved near real-time genomic surveillance allowing the identification of variants [11]. Currently, the Omicron variant has the largest circulation (>64% of genomes) in Botswana. This study sought to characterize SARS-CoV-2 recombinant strains that circulated in Botswana between 2020 and 2023 using 5,254 complete SARS-CoV-2 genomes generated. Furthermore, a mutation profiling analysis was performed to characterize immune escape mutations.

## Materials and methods

### Study ethical considerations

The study was conducted according to the Declaration of Helsinki. The Health Research and Development Committee of Botswana reviewed and approved the protocol (Protocol #HRDC00945; HPDME 13/18/1).

### Study participants, sample collection, and extraction

This is a retrospective analysis of SARS-CoV-2 sequences previously generated during routine national diagnostic testing, surveillance, and sequencing in Botswana. We previously described the criteria for sample selection, extraction, and tiling polymerase chain reaction used in this study [12]. Briefly, residual combined nasopharyngeal and oropharyngeal swabs from COVID-19 diagnosis were routinely and randomly collected each week between September 2020 and September 2023 from all regions of the nine nationwide COVID-19 zones. All samples with a real-time cycle threshold value (qCt) below 32 (qCt ≤32) were selected for sequencing.

### Next-generation sequencing of SARS-CoV-2

SARS-CoV-2 sequencing and analysis were all conducted at Botswana Harvard HIV Reference Laboratory. Next-generation sequencing based on Oxford Nanopore Technologies sequencer GridIONx5 (Oxford Nanopore Technologies, Cambridge, United Kingdom) was used. We previously described the library preparations based on midnight protocol that used to generate the sequences [12].

### SARS-CoV-2 lineage classification

After sequencing, the raw FASTQ sequence output files obtained from the Oxford Nanopore Technologies were processed into consensus FASTA files using reference-based assembly in the genome detective [13]. To minimize spurious results, sequences with an average read depth >500 were considered for downstream analyses. Quality control reports and clades and lineages assignments were done using NextClade [14]. All high-quality genomes with >80 % coverage breadth have been deposited continuously in Global Initiative on Sharing All Influenza Data (GISAID) database [15].

### SARS-CoV-2 recombination analyses

We retrieved 5,254 near–full-length SARS-CoV-2 genomes for Botswana from GISAID generated between 2020 and 2023. The recombination analysis was conducted at the population level using consensus sequences. Rebar (https://github.com/phac-nml/rebar) was used to further assess for recombinant events and annotate the breaking points. The mosaic structure of the putative recombinant was validated using Snipit (https://github.com/aineniamh/snipit). To confirm that recombinants are not laboratory artifacts, the samples of putative recombinants were re-sequenced in duplicates or triplicates in different runs.

### Identifying clusters by phylogenetic analysis

SARS-CoV-2 nucleotide sequences for each lineage were used to generate multiple sequence alignments using NextAlign [14]. Maximum likelihood trees for each lineage were inferred from multiple sequence alignments in IQ-TREE 2 [16] using the best model determined by the jModelTest2 [17]. The trees were annotated in R statistical software version 4.2.2 (R Core Team, R Foundation for Statistical Computing, Vienna, 2022).

### Mutational analysis and assessing for immune escape using *in silico* predictions

Mutations were evaluated relative to their S protein domains, including N-terminal domain_1__3__–30__54_, receptor-binding domain (RBD)_319–541_, heptad repeat 1 (HR1)_912–984_, heptad repeat 2 (HR2)_116__3__-1213_, fusion peptide (FP)_788–806_, transmembrane (TM)_1213-1237_, and cytoplasmic (CT)_1237-1273_. For each recombinant sequence, we assessed lineage-defining mutations based on the S protein using theStanford Coronavirus Antiviral & Resistance Database (COVDB) database [18] to curate additional mutations that co-existed with lineage-defining mutations but whose global prevalence was modest (<5.0 × 10^−5^). Typically, such mutations are flagged as unusual in the COVDB database [18]. For simplicity, these were termed mutations of interest (MutOI).

The 15-mer S peptides—each overlapping by 14 amino acids—were tested for binding to various HLA class I as previously described [10]. The NetMHCpan-4.1 was used to predict binding peptides and their binding affinity scores were categorized based on the log-transformed binding affinity [1 − Log50k (aff)] [19]. Binding epitopes were considered those that satisfy the rank ≤0.5 (EL rank) and score (EL score) ≥0.5 estimations provided by this neural network method. Eight HLA class I alleles were assessed, including *A01:01, *A03:01, *A24:02, *A26:01, *B08:01, *B15:01, *B27:05, and *B40:01.

### Evaluating the impact of MutOI using *in silico* approaches

Mutations were annotated onto a crystal structure of the viral S protein using the Phyre2 tool (http://www.sbg.bio.ic.ac.uk/∼phyre2/html/page.cgi?id=index), with PDB:7DK3 as a scaffold to model amino acid variations in the viral strains. Structural images were visualized in PyMol (Molecular Graphics System, Version 2.0, Schrödinger, LLC).

We used COV2var tool (http://biomedbdc.wchscu.cn/COV2Var/) to gain deeper insights into the properties and impact of MutOI in the S protein. COV2var is a web-based platform that integrates multiple tools to assess (*i*) the physicochemical properties, (*ii*) spatiotemporal distributions of SARS-CoV-2 lineages, (*iii*) positive and negative selection, (*iv*) protein stability, (*v*) IUPred3 for disordered residues, (*vi*) antigenicity of S mutations, (*vii*) immunogenicity of S mutations, and (*viii*) the deep mutational scanning approach for angiotensin converting enzyme (hACE2)-binding affinity and antigenicity.

## Results

**Figure 1:**
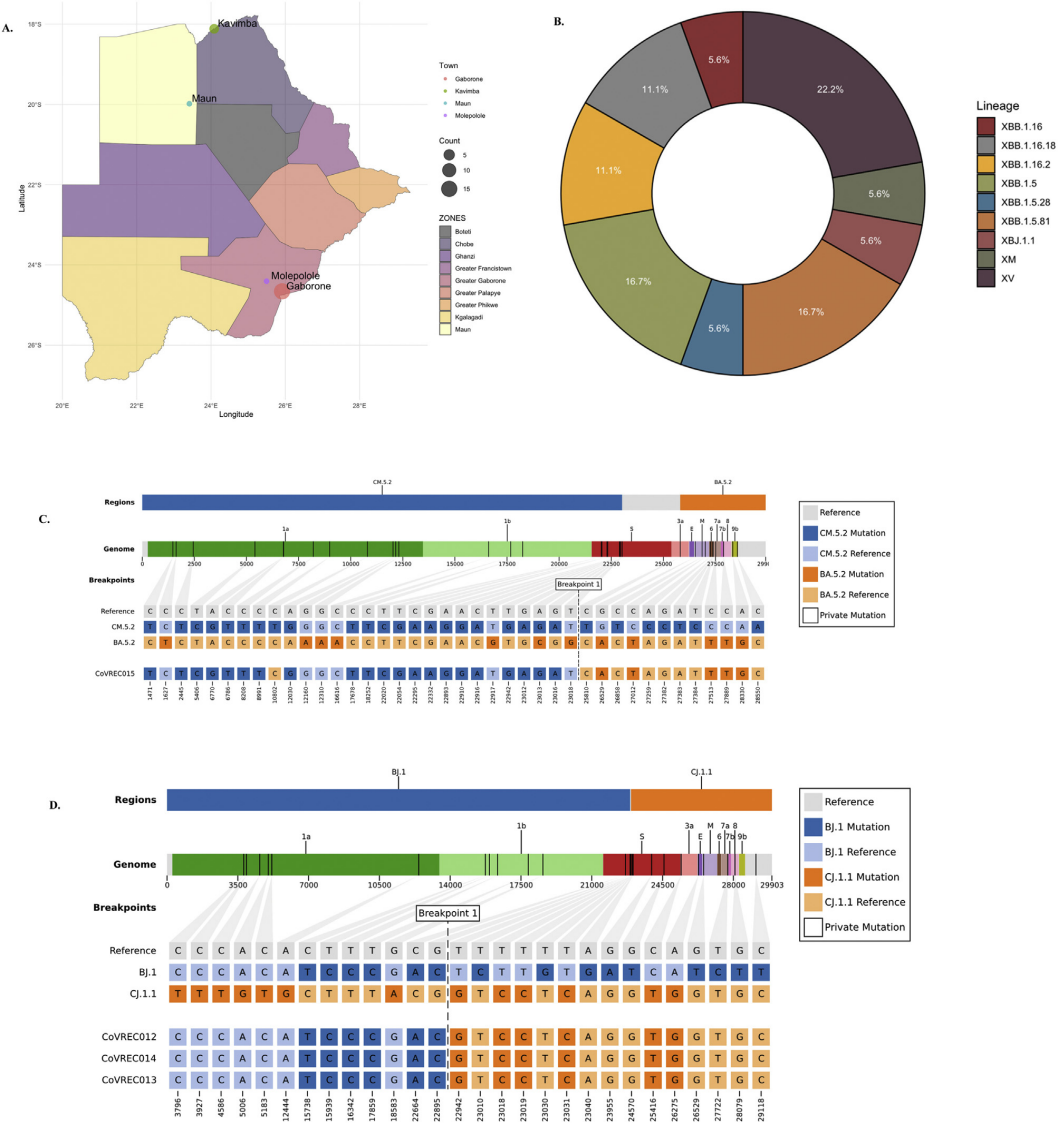
(a) Map of Botswana showing the nine COVID-19 zones and the sample locations. (b) Distribution of putative SARS-CoV-2 recombinant lineages circulating in Botswana from 2020 to 2023. (c) Representative mosaic structures of putative SARS-CoV-2 recombinant sequences characterized in Botswana, XBJ, and (d) XBB.1.5.81.

A total of nine known SARS-CoV-2 recombinant lineages among 20 participants (CoVREC001-15, CoVREC0017-21) were identified in Botswana between 2020 and 2023. The median age was 37 years (Q1, Q3: 19, 53), and 62.0% were female (Table 1a). Females were slightly older, with a median age of 41 years, compared with 37 years for males. Few individuals (n=5) self-reported their COVID-19 vaccination status (Table 1a). About 50% of individuals were symptomatic with influenza-like illnesses (ILIs). No additional information related to comorbidities, such as HIV and hypertension, was available. Of 20 individuals, nine had data on SARS-CoV-2 qCt values for the envelope and ORF genes (Table 1b). The median qCt values were 18.9 (Q1, Q3: 18.24, 22.85) and 25.30 (Q1, Q3: 23.85, 28.10) for the envelope and ORF genes, respectively. All the recombinant sequences were among a subset of COVID-19 samples collected between January 2022 and October 2023. The median collection date of recombinant samples was June 9, 2023 (Q1, Q3: June 1, 2022, June 21, 2023) (Table 1b). Majority of recombinant cases in this study (71.0%) were sampled from Greater Gaborone (Figure 1a). Based on epidemiological data, three individuals had a travel history: one was a returning resident from Zambia (CoVREC018), and the other two were non-residents visiting from China (CoVREC008, CoVREC009) (Table 1b). There were five linked cases, including two involving couples: BWRecoCL001 (CoVREC004, CoVREC005) and BWRecoCL002 (CoVREC002, CoVREC003). BWRecoCL003 involved a mother-infant pair (CoVREC018, CoVREC019), whereas BWRecoCL004 consisted of related travelers (CoVREC008, CoVREC009). The fifth case, BWRecoCL005, was a household cluster in Kavimba, a remote village in the Chobe zone, involving four individuals (CoVREC012-CoVREC014).

**Table 1a:** Characteristics of participants infected with putative SARS-CoV-2 recombinant lineages.

Variable	*n, (%)*
*Gender*Male	19 (34.6%)
Female	36 (65.5%)
*Age in years, median (Q1, Q3)*	40 (18.5, 53)37 (18, 57.5)41 (19, 53.0)
OverallMale	
Female	
*Symptoms at onset*	*n, (%)*
Yes	11 (55.0%)
No	6 (30.0%)
Not disclosed	3 (15.0%)
*Reason for testing*	*n, (%)*
Point of entry:	1 (5.00%)
Routine testing/contact tracing:	8 (25.0%)
Symptoms:	11 (55.0%)
*Lineages:*	*n, (%)*
XBB-like	2 (10.0%)
XBB.1.16.	1 (5.0%)
XBB.1.16.18	2 (10.0%)
XBB.1.16.2.	2 (10.0%)
XBB.1.5	3 (15.0%)
XBB.1.5.28	1 (5.00%)
XBB.1.5.81	3 (15.0%)
XBJ.1.1	1 (5.00%)
XM	1 (5.00%)
XV	4 (20.0%)
*Vaccination status (n = 20)*	*n, (%)*
Vaccinated	6 (30.0%)
Not disclosed	14 (70.0%)
*Vaccine name (N = 6)*	*n, (%)*
AstraZeneca	2 (33.3%)
Sinovac	3 (50.0%)
Unspecified	1 (16.7%)

n, sample size; Q_1_, lower quartile range; Q_3_, upper quartile range; %, percentage.

**Table 1b:** Characteristics of study participants with recombinant SARS-CoV-2 samples.

Participant ID	Date	Age	Sex	Lineage	location	N_gene_	ORF_gene_	Symptomatic	Comments	Fully Vaccinated	Product
CoVREC001	2022-01-12	53	M	XBB-like	Gaborone			ND	Routine Surveillance		
CoVREC002	2023-06-18	62	M	XBB.1.16.2	Gaborone	22.6		✔	Symptomatic (fever, sore throat, cough, headache)		
CoVREC003	2023-06-18	41	F	XBB.1.16	Gaborone	23.1	28.1	✔	Fever, Sore throat, Cough, Headache)		
CoVREC004	2023-10-08	70	F	XBB.1.16.18	Gaborone			No		✔	AstraZeneca
CoVREC005	2023-10-08	81	M	XBB.1.16.18	Gaborone			No		✔	AstraZeneca
CoVREC006	2022-05-23	53	F	XBB-like	Gaborone			ND	Routine Surveillance		
CoVREC007	2023-06-19	39	F	XBB.1.16.2	Gaborone	19.2	23.8	✔	Symptomatic (fever, sore throat, cough, headache)		
CoVREC008	2023-01-15	24	M	XBB.1.5	Gaborone			No			
CoVREC009	2023-06-01	37	M	XBB.1.5	Gaborone	27.3	25.3	✔	Sore throat, Cough, Headache, Fatigue	✔	Sinovac
CoVREC010	2023-06-20	53	F	XBB.1.5	Gaborone			✔	Symptomatic (cough, painful chest)	✔	Sinovac **
CoVREC011	2023-08-24	14	F	XBB.1.5.28	Molepolole			ND	Routine Surveillance		
CoVREC012	2023-06-21	19	F	XBB.1.5.81	Kavimba			✔	Symptomatic		
CoVREC013	2023-06-21	44	F	XBB.1.5.81	Kavimba			✔	Symptomatic		
CoVREC014	2023-06-21	12	M	XBB.1.5.81	Kavimba			✔	Symptomatic		
CoVREC015	2023-01-23	33	F	XBJ.1.1	Gaborone			No	Asymptomatic	✔	
CoVREC017	2022-01-12	73	F	XM	Maun	23.94	26.45	No	Asymptomatic, POE		
CoVREC018	2022-04-12	1 M	M	XV	Gaborone	13.33	20.96	✔	Fever, vomiting, Hospitalization		
CoVREC019	2022-04-13	17	F	XV	Gaborone	21.19	27.52	No	Asymptomatic		
CoVREC020	2022-06-04	16	F	XV	Gaborone	18.57	23.85	✔	Sore throat, Cough, Headache, Sneezing, Mouth sores)		
CoVREC021	2022-06-16	46	F	XV	Gaborone	11.74		✔	Fever, Cough, Headache)	✔	Sinovac

### Detection of putative recombination in Botswana

After removing duplicate sequences, the prevalence of SARS-CoV-2 recombinant lineages was ∼0.40% (20 of 5,152). We found nine different putative recombinants, including XBB.1.16, XBB.1.16.18, XBB.1.16.2, XBB.1.5, XBB.1.5.28, XBB.1.5.81, XBJ.1.1, XM, and XV, with 57.0% being descendants of XBB, as shown in Figure 1b. Overall, the immune escape values for the XBB* lineages were higher than for recombinants XV and XM. The relationship between immune escape and hACE2 binding are shown in Figure S1. The recombination event breaking points are situated from the tail of the S protein to the 3’ end of the genome in the RBD for 10 lineages, as shown in Table 2. The identified inter-VOC recombinant sequences primarily consisted of the BA.1* and the BA.2* for XBB* the inter-Omicron recombinants. The inferred mosaic genome structures of four SARS-CoV-2 recombinants are annotated in Figures 1:c and d and others in Figure S2.

**Table 2:** Summary of detected SARS-CoV-2 recombinant lineages, depth of coverage, breaking points, and mutations of interest.

Recombinant lineage	Isolate name	Replicates	Average read depth^†^	Total reads	Major parent	Breaking points	SARS-CoV-2 domain	Cluster transmission	MutOI	Global frequency (N)
XBJ.1.1	*CoVREC015*	1	1768.50	80,985	XBJ+ BA.5.2	S:23019 – 25809	S1/S2 cleavage site to S2 domain	-	nsp3_C118Y N_D3N	1,241 457
XBB.1.16.2	*CoVREC007*	1	1366.80	70,933	BJ.1+ BA.2.75			-	nsp3_P108H nsp3_V573I	7,666 1,334
XBB.1.16.18	*CoVREC004*	1	970	39,400	BJ.1+ CJ.1	S: 22897 – 22941	RBD	**BWRecoCL001**	ORF6: D61L	3
	*CoVREC005*	1	599	72,00	BJ.1+ CJ.1	S: 22897 – 22941	RBD	**BWRecoCL001**		
XBB.1.16	*CoVREC003*	6	343.1	19,828	BJ.1+ CJ.1	S: 22897 – 22941	RBD	**BWRecoCL002**		
	CoVREC002*	5	246	12,621	BJ.1+ CJ.1	S: 22897 – 22941	RBD	**BWRecoCL002**		
XBB.1.5.81	*CoVREC013*	1	846.4	25,057	BJ.1+ CJ.1	S:22896 – 22941	RBD	**BWRecoCL0045**	3CLpro: Q110* nsp13: H290Q S: P1162A S: D1163P N: P80L	3,246 216 2,742 41 1,193
	*CoVREC012*	4	538.2	12,849	BJ.1+ CJ.1	S:22896 – 22941	RBD	**BWRecoCL0045**	-	-
	*CoVREC014*	1	1400	>100,00	BJ.1+ CJ.1	S:22896 – 22941	RBD	**BWRecoCL0045**	-	-
XBB.1.5.28	*CoVREC011*	1	1350	34,520	BJ.1+ CJ.1	S:22897 – 22941	RBD		RdRp:V233I nsp3_A231T	1,819 562
XBB.1.5	*CoVREC009*	2	573.2	26,473	BJ.1+ BA.2.75	S: 22897 – 22941	RBD	**BWRecoCL004**		
	*CoVREC008*	1	1621.20	77,099	BJ.1+ BA.2.75	S: 22897 – 22941	RBD	**BWRecoCL004**		
	*CoVREC010*	1	903.9	49,709	BJ.1+ BA.2.75	S: 22897 – 22941	RBD	**-**	S:Q474K S:E990D	274 802
XBB-like	*CoVREC001*	1	458	20,790	BJ.1+ BA.2.75	S:22897 – 22941		**-**	-	-
	*CoVREC010*	1						**-**	-	-
XM	*CoVREC017*	1	796.1	35,063	BA.1+ BA.2.60	S: 21847 – 22672 S: 23203 – 24129 S: 24948 – 26857	NTD S1/S2 cleavage site S2 domain	**-**	nsp9: I65M	8
XV	*CoVREC018*	1	1099.00	>100,000	BA.1.18+ BA.2.48	ORF1a: 1524 – 15713	nsp3/nsp4	**BWRecoCL003**	ORF3a:E261G	1,798
	*CoVREC019*	1	1700.00	1099.00	BA.1.18+ BA.2.48	ORF1a: 1524 – 15713	nsp3/nsp4	**BWRecoCL003**		
	*CoVREC021*	1	1071.50	46,347	BA.1.18+ BA.2.48	ORF1a: 15241–17409	nsp3	**-**	-	-
	*CoVREC020*	1	1666.30	80,187	BA.1.18+ BA.2.48	ORF1a: 1524 – 15713	nsp3/nsp4			

FCS, furin cleavage site; NTD, N-terminal domain; nsp, non-structural protein; ORF, open reading frame; RBD, receptor-binding domain; S1, spike subunit 1; S2, spike subunit 2.

### Transmission patterns of recombinant lineages based on epidemiolocal data

Based on the epidemiological data obtained during sample collection, the earliest known recombinant case was XM obtained in January 2022 in Gaborone. Overall, the median time to detect SARS-CoV-2 recombinant lineages in Botswana relative to the first global report was 104 days (Q1; Q3: 55; 147), as shown in Supplementary Figure S3A. Individual temporal graphs showing sampling dates vs counts are provided in Supplementary Figure S3B. The XV and XM circulated predominantly during 2022. The XBB.1.5-like and XBB.1.6-like lineages co-circulated mostly in 2023 at the same time (Figure S3A). The comparison of sample collection of recombinant sequences and global sequences are shown in Figure S5.

One case of XBJ.1.1 (CoVREC018) was identified in January 2023 from an individual who had a vaccination history (July 2022) and an unknown travel history (Figure S4A). As shown in Table 1b, other singular cases were identified from a symptomatic individual (CoVREC007) infected with XBB.1.16.2 and had undisclosed travel history. The XM case (CoVREC018) had a travel history and tested positive for COVID-19 at the Maun port of entry between Botswana, Namibia, and Zambia. Also, this is the only known case of XM in Botswana. The individual was asymptomatic and the qCt value was 23.94 for the N gene.

Four cases of XV (CoVREC018-CoVREC021) were identified in Gaborone between April and June 2022. The median qCt values were 15.95 for the N gene and 23.85 for the ORF gene. Three participants were young (1 month, 16 years, and 17 years old) and had influenza-like illness, whereas a 46-year-old fully vaccinated woman also showed symptoms. Two cases were linked (mother-infant transmission), with the infant hospitalized for severe symptoms.

In January 2023, two XBB.1.5 cases from a travel-related cluster were the first reported in Botswana, followed by a larger household cluster (BWRecoCL005) of in Kavimba in the Chobe district bordering Namibia. Other clusters (BWRecoCL001 and BWRecoCL002) included elderly asymptomatic and middle-aged symptomatic individuals, with no travel history provided.

### Tracing possible origins of variants based on phylogenetic analysis

**Figure 2:**
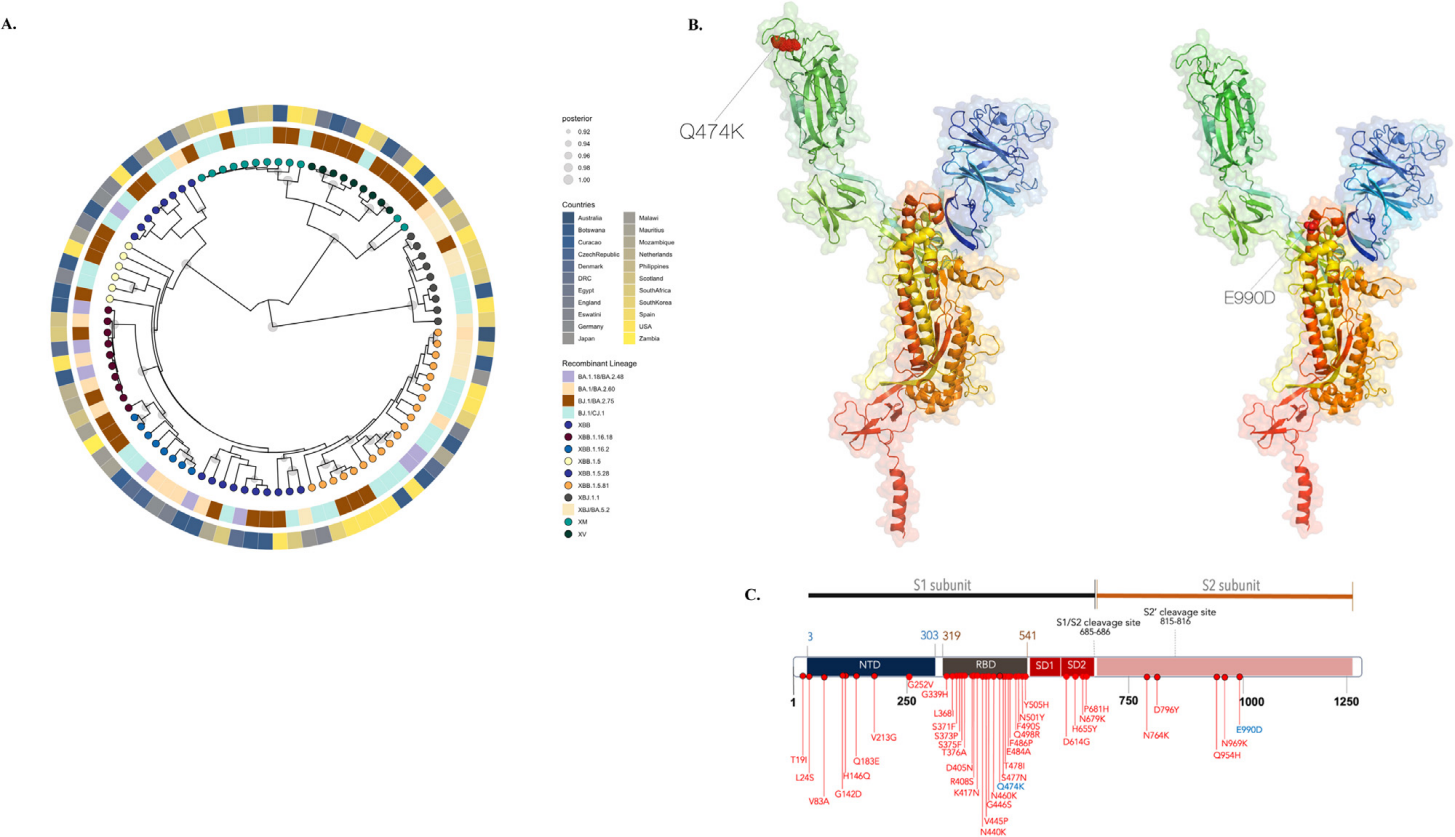
(a) Maximum likelihood phylogenetic trees summarizing the clustering of 20 recombinant sequences from Botswana and 72 closely related representative reference sequences with posterior probability ≥0.90. Trees were annotated based on circular topology. (b) The 3D structure of spike protein. The crystal structures of SARS-CoV-2 Spike annotated to surface topology using PyMOL3. (c) Deleterious mutations are highlighted in blue. XBB.1.5 classical mutations: T19I, L24S, V83A, G142D, H146HQ, Q183E, V213E, G252V, G339H, R346T, L368I, S371F, S373P, S375F, T376A, D405N, R408S, K417N, N440K, V445P, G446S, N460K, S477N, T478I, E484A, F486P, F490S, Q498R, N501Y, Y505H, D614G, H655Y, N679K, P681H, N764K, D796F, Q954H, and N969.

A maximum likelihood-based phylogenetic inference supported by high posterior probability [pp] ≥0.90 is shown in Figure 2a. The tree was based on 20 sequences from Botswana and 72 global reference sequences obtained from GISAID (EPI_SET_240822bm; 10.55876/gis8.240822bm). Similar to the NextClade analysis, the SARS-CoV-2 sequences from Botswana clustered with respective sequences from nine lineages.

Based on GISAID data, all the SARS-CoV-2 recombinant lineages we report in this study (except for XBB.1.5 and XBB.1.16) had a low global prevalence of <0.0001%, with only 2,960 cases ever reported in Africa based on GISAID dataset (Table S1). Only 10 recombinant cases including XBJ.1.1 (n = 1), XV (n = 4), and XM (n = 5) were recorded in Africa during the time of this study, of which XBJ.1.1 and XV were only reported in Botswana. The XBJ.1.1 sequence from Botswana*—CoVREC015*—clustered closely (pp >90) with South Korean sequences sampled between January and February 2022 (Figure 2a, Figure S4.A). The root-to-tip regression obtained from TempEst analysis for all the XBJ.1.1 lineages showed a relatively strong clock-like behavior (correlation coefficient = 0.56) (Figure S4.B). Similarly, the *CoVREC017* sequence was analyzed against all global XM sequences, and it clustered closely with regional sequences (Namibia, South Africa) (pp >0.90) (Figure S4C). Notably, sequences in the respective clusters, BWRecoCL001-5, showed high genetic relatedness supported by pp ≥0.90.

### Mutational spectrum, mutations of interest, and their impact on immune escape

As shown in Table 3a, we identified 16 MutOI, including eight in ORF1ab, four in the S protein, two in the N protein, and one each in ORF3a and ORF6. All were single observations, except for ORF3a:E261G and ORF6:D61L, each of which appeared in two samples. Among the 16 mutations, seven (43.8%) were deleterious (Table 3a). Among the four mutations in the S protein, S:Q474K was the only mutation within the RBD region. Because the RBD is responsible for viral attachment to hACE2 and promoting fusion with the epithelial cell membrane, we examined the S:Q474K mutation in more detail. The S:Q474K demonstrated CTL escape capabilities in the epitope YQAGSTPCNGVEGF and HLA types *A0301, *A2402, *B0801, and *B1501. This disruption of HLA epitope binding mirrors the behavior of previously characterized immune escape mutations, such as F486P in STPCNGVEGPNCYF, G339H→ FHEVFNATRFASVY, L368I →KRISNCVADYSVIY, Q183E →KEGNFKNLREFVFK, R346T →TTFASVYAWNRKRI, V213E →KIYSKHTPINLER, and V445P →KPGGNYNYLYRLFR (Table 3b). S:Q474K was characterized from CoVREC01, an XBB.1.5 sequence, that had 11 additional mutations in S protein, including V83A, Q183E, and V213E in the N-terminal domain; G339H, R346T, L368I, V445P, and F486P in the S2; Q474K in the RBD; D796F in the heptad repeat 1; and E990D in the heptad repeat 2 region (Figure 2b-c).

**Table 3a:** Summary of mutations of interest and the SARS-CoV-2 protein they are located.

Gene	Mutation	Protein	Specific domain	*n*	AA impact	Global prevalence(∼16.8 million)
ORF1ab	P108H	nsp3_1945_	Ubl1	1	Deleterious	4.57×10^−4^
	C118Y	nsp3_1945_	Ubl1	1	Neutral	7.39×10^−5^
	A231T	nsp3_1945_	ADRP (Mac1)	1	Neutral	3.35×10^−5^
	V573I	nsp3_1945_	near SUD	1	Neutral	7.94×10^−5^
	Q110^a^	Nsp5_306_	M^pro^/3CL^pro^	1	-	1.93×10^−4^
	I65M	nsp9_113_	nsp9	1	Deleterious	4.76×10^−7^
	V233I	nsp12_932_	RdRp	1	Neutral	1.08×10^−4^
	H290Q	nsp13_601_	Helicase 1B	1	Deleterious	1.29×10^−5^
ORF3a	E261G	ORF3a_275_	Ion domain	2	Neutral	1.07×10^−4^
ORF6	D61L	ORF6_61_	Interferon antagonist domain	2	Deleterious	1.79×10^−7^
N	D3N	N_419_	NTD	1	Neutral	2.72×10^−5^
	P80L	N_419_	NTD	1	Deleterious	7.10x10^-5^
S	Q474K	S_1273_	RBD	1	Neutral	1.63×10^−5^
	E990D	S_1273_	S2	1	Neutral	4.78×10^−5^
	P1162A	S_1273_	S2	1	Deleterious	1.63×10^−6^
	D1163P	S_1273_	S2	1	Deleterious	2.44×10^−6^

a Denotes stop codon. ADRP, ADP-ribose phosphatase domain; Mac1, macrodomain; Mpro, main protease; N, nucleocapsid protein; NTD, N-terminal domain; ORF, open reading frame; RBD, receptor-binding domain; RdRp, RNA dependent RNA polymerase; S, surface protein; S1, sub-unit 1; S2, sub-unit 2; SUD, SARS-unique domain; UbI, ubiquitin-like domain 1; 3CLpro, 3C-like protease.

**Table 3b:** Predicted SARS-CoV-2 epitopes in spike protein restricted to HLA class I molecules.

AA position	Mutation	HLA class I	Reference epitope	Mutated epitope
183	Q183E	A0301	KQGNFKNLREFVFK	KEGNFKNLREFVFK
213	V213E	A0301B2705	KIYSKHTPINLVRD; VRDLPQGFSALEPL	KIYSKHTPINLERD; ERDLPQGFSALEPL
339	G339H	A0101B4001	FGEVFNATRFASVY; PFGEVFNATRFASV	FHEVFNATRFASV; YPFHEVFNATRFASV
346	R346T	B2705A0301	TRFASVYAWNRKRI; ATRFASVYAWNRKR	TTFASVYAWNRKRI; ATTFASVYAWNRKR
368	L368I	A0101A0301B2705	SNCVADYSVLYNSA; KRISNCVADYSVLY; KRISNCVADYSVLY	SNCVADYSVIYNSA; KRISNCVADYSVIY; KRISNCVADYSVIY
445	V445P	A0301	KVGGNYNYLYRLFR	KPGGNYNYLYRLFR
474	**Q474K**	A2402B1501	YQAGSTPCNGVEGF; YQAGSTPCNGVEGF	YKAGSTPCNGVEGF; YKAGSTPCNGVEGF
489	F486P	A0101A2601B1501B4001	STPCNGVEGFNCYF; *STPCNGVEG**F**NCYF; YQAGSTPCNGVEGF; CNGVEGFNCYFPLQ	STPCNGVEGPNCPF; STPCNGVEGPNCPFY; QAGSTPCNGVEGP; CNGVEGPNCYFPLQ

HLA, human leukocyte antigen.

## Discussion

In this study, we identified the SARS-CoV-2 recombinant lineages XBB.1.5, XBB1.5.81, XBB.1.5.28, XBB.1.6, XBB.1.6.18, XBB.1.6.2, XBJ.1.1, XV, and XM among the 5,254 complete genomes generated in Botswana from 2020 to 2023. To the best of our knowledge, this is the first study to document cases of XBJ.1.1 and XV in Africa. The earliest recombinant strains were detected in January 2022 in Botswana, a few weeks after the first reports of the Omicron VOC and its other sub-lineages [20]. Overall, the most recombinants were descendants of Omicron sub-lineages (XBB*), a trend that has been observed regionally and globally (Supplementary Table 1). This is likely due to prolonged high co-circulation of succeeding emerging Omicron lineages [6]. The XBJ.1.1 lineages predominantly circulated in Asia, of which <80 global sequences have been reported (EPI_SET_240619un, 10.55876/gis8.240619un).

XBB.1.5-like variants had the highest prevalence (35%) in this study. The XBB.1.5 is a descendant of XBB.1 with a S:F486P mutation and a translational stop in ORF8 (ORF8:G8stop) resulting from MHC suppression [21]. XBB.1.5 and its subvariants (e.g. XBB.1.5.81 and XBB.1.5.28) have been associated with increased binding affinity to the hACE2 receptor, transmissibility, infectivity, and immune resistance [22]. XBB.1.5 was first characterized in October 2022 in the United States and listed as a VOI by the WHO in March 2023 [23]. The XBB.1.5.81 has an extra S:K478R and has been predominately reported in South Africa [24]. The XBB.1.5.28 is a descendant of XBB.1.5 with an extra S:K478R on 17124C polytomy that was reported primarily in the Americas and not well-known in Africa [24].

The main hotspot of breaking points was the RBD in the S subunit 1. Our results corroborate with other studies that have indicated that most of the emerging SARS-CoV-2 recombinant sequences have breaking points in the S protein and are mainly associated with immune escape [3]. CD8+ T-cell immunity to SARS-CoV-2 has been implicated in COVID-19 severity and virus control [25]. However, recombination, together with the emergence of nonsynonymous mutations in MHC-I–restricted CD8+ T-cell epitopes, facilitate SARS-CoV-2 immune escape [6]. Several studies have utilized bioinformatics approaches to characterize SARS-CoV-2 mutations in MHC-I–restricted epitopes that may evade CD8+ T-cell responses. This approach is desirable because it provides a cost-effective, rapid, and alternative way to screen mutations. Candidate mutations will be evaluated further *in vitro* in future studies.

In this study, we used *in silico* analyses to characterize the potential impact of unusual mutations among the 20 recombinant sequences. We identified rare and potentially deleterious mutations at various sites in five SARS-CoV-2 proteins—ORF1a, ORF6, ORF3a, N, and S—that could impact the virus. Notably, S:Q474K exhibited properties of CTL-mediated immune escape. The mutations reported in this study warrant follow-up *in vitro* investigation. S:Q474K was identified from an XBB.1.5 case (CoVREC010) who was fully vaccinated with Sinovac. This mutation is rare, appearing in only 247 sequences on GISAID, with ∼9.5% being XBB.1.5 (Supplementary Table 1). Our results are similar to a previous study that reported the emergency of escape mutations and resistance mutations in an immunocompromised patient infected with a Delta-Omicron recombinant variant [3]. To validate our finding, we compared S:Q474K with the other seven previously characterized mutations shown in Table 3b. S:Q474K expressed similar immune escape properties with the potential ability to escape the CD8+ T-cell–mediated immunity when previously characterized epitopes—YKAGSTPCNGVEGF_473-486_, and YQAGSTPCNGVEGF_473-486_—were analyzed for binding with different HLAs [26]. Overall, the binding free energy change of S:Q474K was estimated at 0.1404 kcal/mol, suggesting a weak positive binding between S protein and hACE2 [27]. Additional *in vitro* analysis is warranted to gain a further understanding of this mutation.

We also identified another relatively rare mutation, N:S37P occurring in the N-terminal intrinsically disordered region of N protein from an XBBJ.1.1 sequence. The XBBJ.1.1 sequence we report here is the only case harboring N:S37P among the publicly available XBJ.1.1 sequences (10.55876/gis8.240619un). N:S37P occurred in <0.01% of all global SARS-CoV-2 sequences as of June 2024. Interestingly, the N:S37P mutation was characterized in the N protein of RaTG13 sequence, a bat coronavirus that exhibits up to 96% genetic similarity with SARS-CoV-2 [28]. Among the >17 million SARS-CoV-2 sequences publicly available, N:S37P has been reported mostly in Omicron (n = 5,395) compared with other lineages such as Alpha (n = 1,651), Beta (n = 4), and Gamma (n = 51), (10.55876/gis8.240619un). Because residue 37 is in the intrinsically disordered region that is not part of a structured domain, we did not evaluate its impact on immune escape. However, future studies are warranted to evaluate its impact on the structural flexibility of the protein.

We investigated transmission patterns using phylogenetic inferences, alongside characterizing multiplicity of infection and studying the genomic epidemiology of recombinant variants. Most epidemiologically linked SARS-CoV-2 cases showed low divergence, with 0-2 mutations observed in nearly full-length viral sequences. Three XBB.1.5.81 cases, detected during a household cluster investigation in Kavimba, Botswana (BWRecoCL005) in Namibia, were among the 199 global sequences reported on GISAID (EPI_SET_240619ot; 10.55876/gis8.240619ot) by June 19, 2023, with 61% from southern Africa (South Africa, Namibia, Botswana, and Eswatini).

A unique XBJ.1.1 case in Botswana (CoVREC015) clustered with South Korean sequences, suggesting a transmission link despite no recorded travel history. The Botswana and South Korean sequences were sampled within the same period (January and February 2022).

XBB.1.16.18 cases were identified in an elderly vaccinated couple, part of 956 global cases. Phylogenetically, the XBB.1.16.18 cases clustered closely with sequences from the United States, supported by high bootstrap *P* ≥0.90. XM involved a 73-year-old patient from Zambia and over 580 global XM cases have been reported.

Our study had several limitations. We lacked sufficient travel histories and immediate contact data to trace transmission routes in many cases. COVID-19 testing and sequencing in Botswana significantly declined after the WHO ended the Public Health Emergency of International Concern on May 5, 2023, which affects the interpretation of our findings. Detecting emerging recombinants in routine surveillance is also difficult due to their lower fitness than dominant strains [3]. In addition, our study was cross-sectional, focusing only on population-level recombinant analysis. Investigating intra-host recombinants could have revealed more variants, especially in immunocompromised patients, in whom co-infection with multiple variants can drive viral recombination [29]. However, detecting these intra-host events requires extensive genome curation and sequencing analysis. For future research, we recommend longitudinal studies with sufficient clinical data to better understand the impact of emerging mutations.

Overall, tracking the evolution of SARS-CoV-2, especially recombinant variants, is critical because they may weaken vaccine effectiveness and detection methods [30]. To the best of our knowledge, this study is the first to characterize SARS-CoV-2 recombinant lineages in Botswana, identifying mutations that could promote immune escape and facilitate virus spread. These mutations, such as those in other studies, are linked to reduced vaccine efficacy [3]. For instance, high prevalence of breakthrough infections driven by XBB-like variants has led to the development of new vaccines, such as those using the monovalent JN.1 lineage as the antigen.

## Conclusion

We identified nine SARS-CoV-2 recombinant lineages from the largest SARS-CoV-2 data set generated in Botswana from 2020 to 2023. The majority were descendants of XBB*, resulting from the recombination between two sub-lineages of the VOC Omicron. We identified and characterized low-level mutations such as S:Q474K in the S protein that may impact CTL immune escape. The mutations identified in this study were investigated using *in silico* analyses only; therefore, follow-up *in vitro* assays are required to validate these findings. Our findings underscore the critical need for ongoing and vigilant genomic surveillance to monitor the emergence of new mutations and variants.

## Declarations of competing interest

The authors declare no conflicts of interest. The funders had no role in the design of the study; in the collection, analyses, or interpretation of data; in the writing of the manuscript; or in the decision to publish the results.

## Appendix Supplementary materials

Image, application 1
