# Supplementary material for: Genomic epidemiology and immune escape of SARS-CoV-2 recombinant strains circulating in Botswana
Source: IJID Reg. 2024 Nov 2;13:100484. doi: 10.1016/j.ijregi.2024.100484 (PMC11636131; doi:10.1016/j.ijregi.2024.100484)
Supplement: Supplementary file 1 [file mmc1.docx]

**Supplementary Table S1:** Overall information on recombinants we report in this study. Information of regional and global prevalence is provided.

| Recombinant | Additional Mutations | BW | | AFR | | Global | | First  Reported | | First SAMpling  in BW | | First  Reports | | First Case  (collection) | | Last Reports | | Last  Reported | | Time in Circulation | |
| --- | --- | --- | --- | --- | --- | --- | --- | --- | --- | --- | --- | --- | --- | --- | --- | --- | --- | --- | --- | --- | --- |
| XBB | - | 2 | 246 | | 5,107 | | India | | 2022-Dec-18 | | 2022-Sep-09 | | 2022-Aug-13 | | 2024-Mar-04 | | Chile | | 569 | |  |
| XBB.1.16 | S:E180V, S:478R | 9 | 454 | | 39,481 | | Malaysia | | 2023-May-17 | | 2022-Dec-21 | | 2022-Nov-23 | | 2024-May-03 | | USA | | 527 | |  |
| XBB.1.16.18 | ORF7a:T39I, | 2 | 4 | | 956 | | USA | | 2023-Aug-17 | | 2023-Mar-20 | | 2023-Mar-13 | | 2024-Feb-17 | | England | | 341 | |  |
| XBB.1.16.2 | ORF3a:V13L, ORF1a:P926H, | 5 | 74 | | 3,453 | | India | | 2023-Jul-06 | | 2023-Feb-24 | | 2023-Feb-13 | | 2024-Jan-28 | | USA | | 349 | |  |
| XBB.1.5 | S:F486P | 6 | 1,768 | | 188,310 | | India | | 2022-Dec-06 | | 2022-Oct-10 | | 2022-Sep-14 | | 2024-Apr-12 | | Canada | | 576 | |  |
| XBB.1.5.28 | S:K478R, on 17124C polytomy, | 1 | 11 | | 1,061 | | USA | | 2023-Aug-24 | | 2023-Jan-20 | | 2022-Dec-26 | | 2024-Feb-18 | | USA | | 419 | |  |
| XBB.1.5.81 | S:478R, G4657A, | 7 | 121 | | 198 | | Chile | | 2023-Jun-21 | | 2023-Mar-04 | | 2023-Feb-14 | | 2023-Oct-24 | | Mexico | | 252 | |  |
| XBJ.1.1 | S:G485D, N:R195K, | 1 | 1 | | 71 | | Philippines | | 2023-Jan-30 | | 2022-Dec-06 | | 2022-Oct-28 | | 2023-Apr-10 | | Korea | | 164 | |  |
| XM | - | 2 | 6 | | 585 | | India | | - | | 2022-Jan-17 | | 2022-Jan-01 | | 2022-Aug-28 | | USA | | 239 | |  |
| XV | - | 4 | 4 | | 42 | | Denmark | | 2022-Apr-02 | | 2022-Feb-08 | | 2022-Jan-31 | | 2022-Apr-27 | | Italy | | 86 | |  |

**^
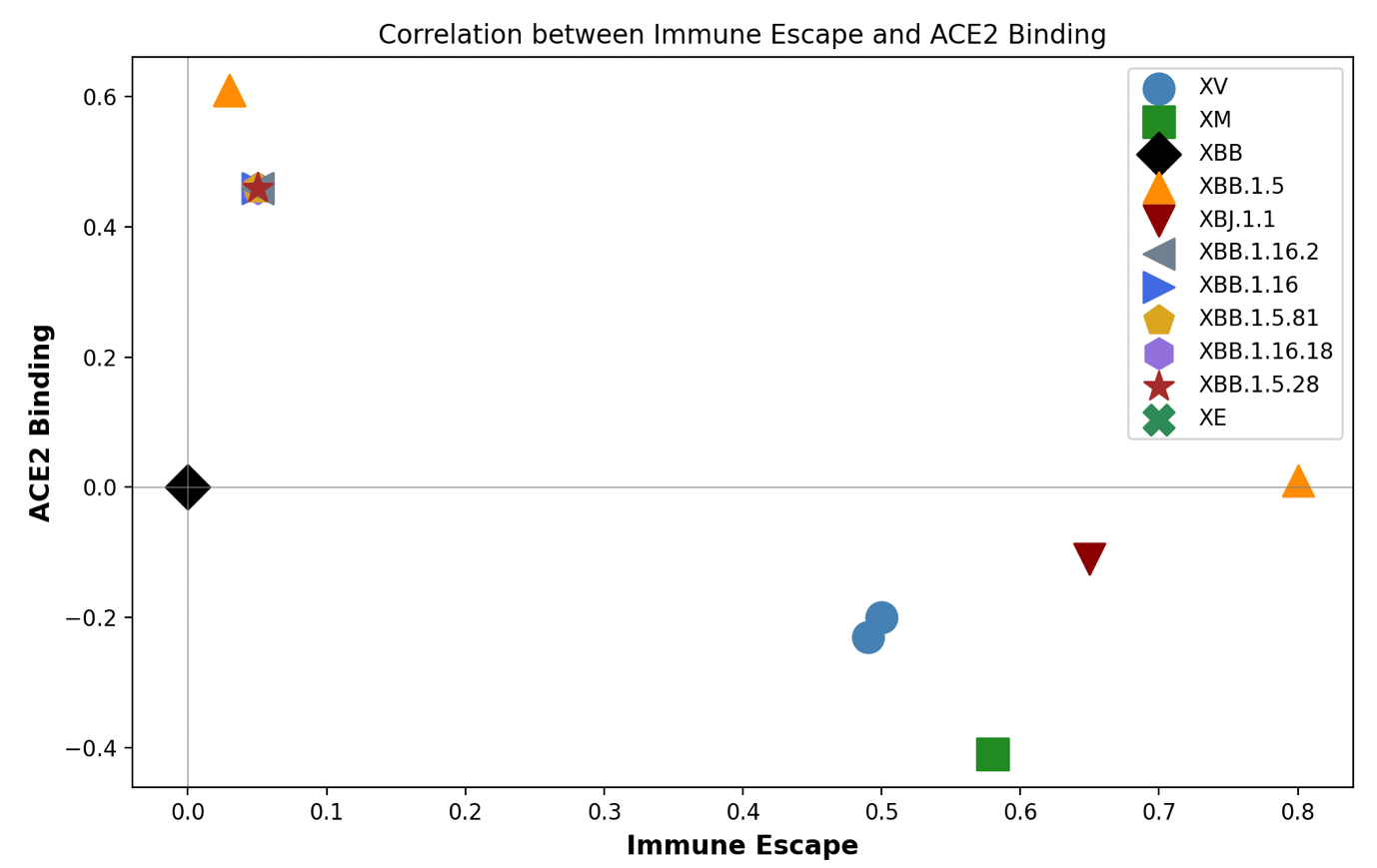
^**

**Figure S1:** Scatterplot of ACE2 binding versus immune escape estimated values. The plot is bases on valued calculated by Bloom lab's ACE2 binding calculator and Bloom lab's antibody escape calculator, respectively.


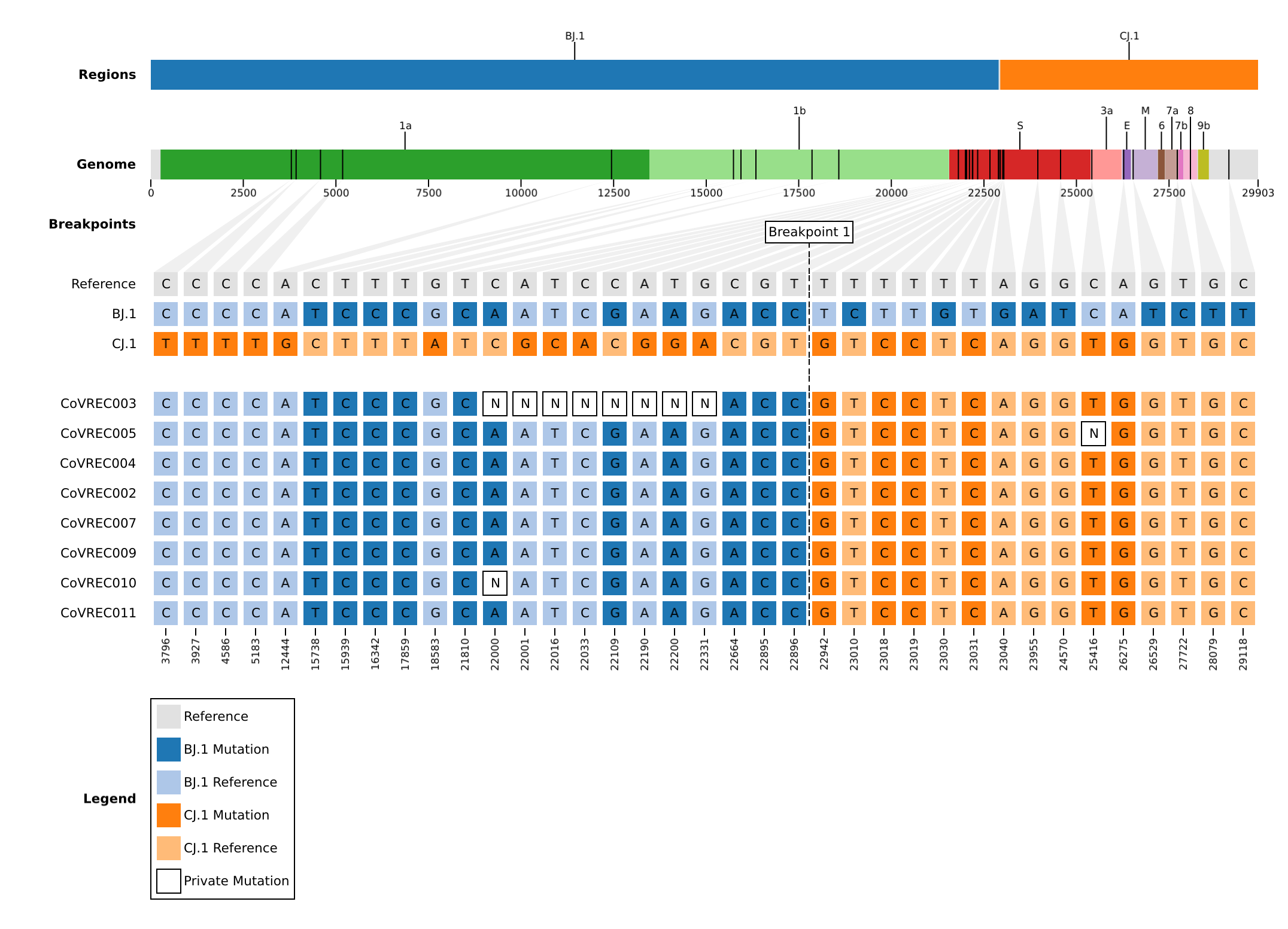


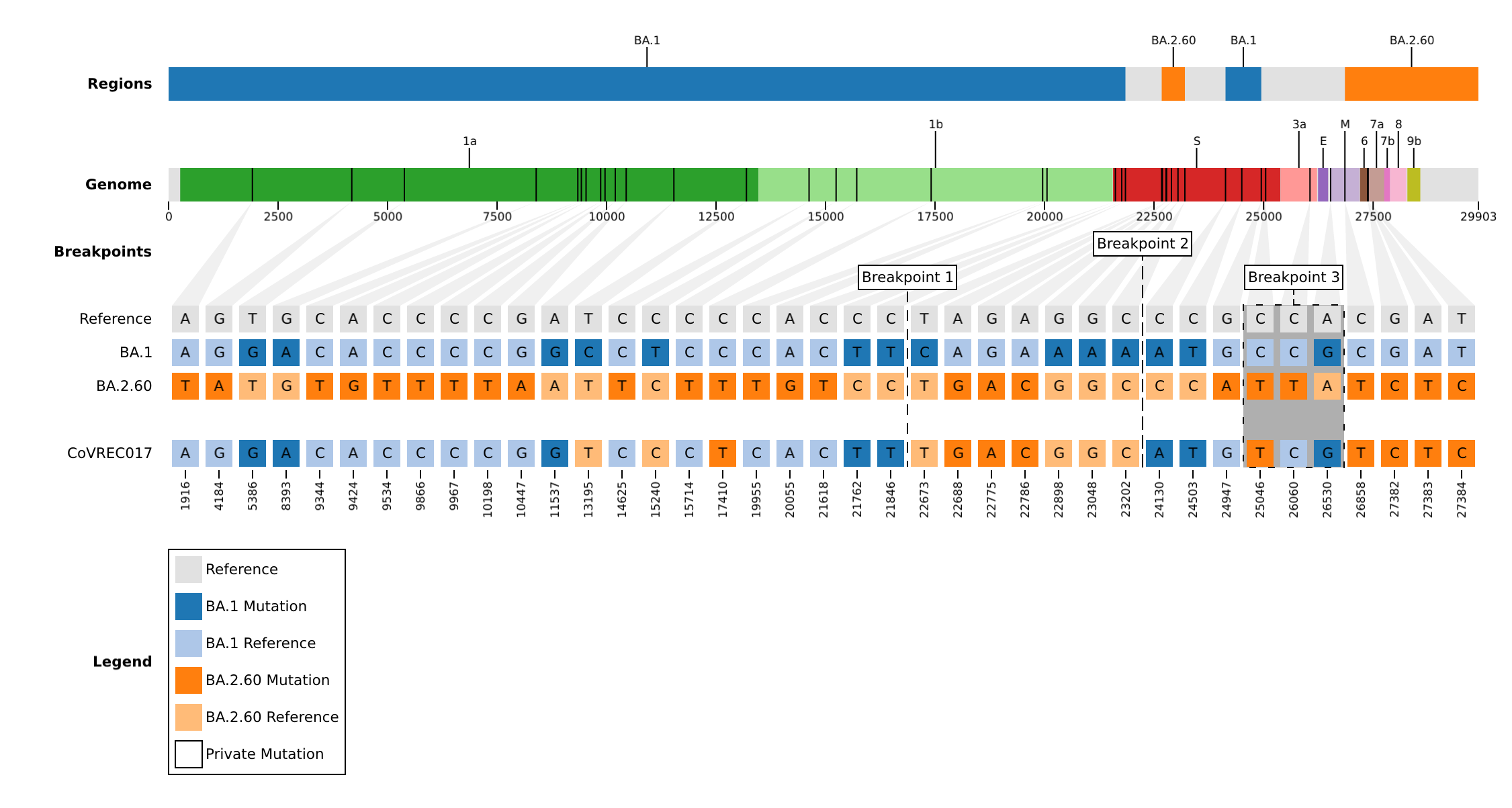


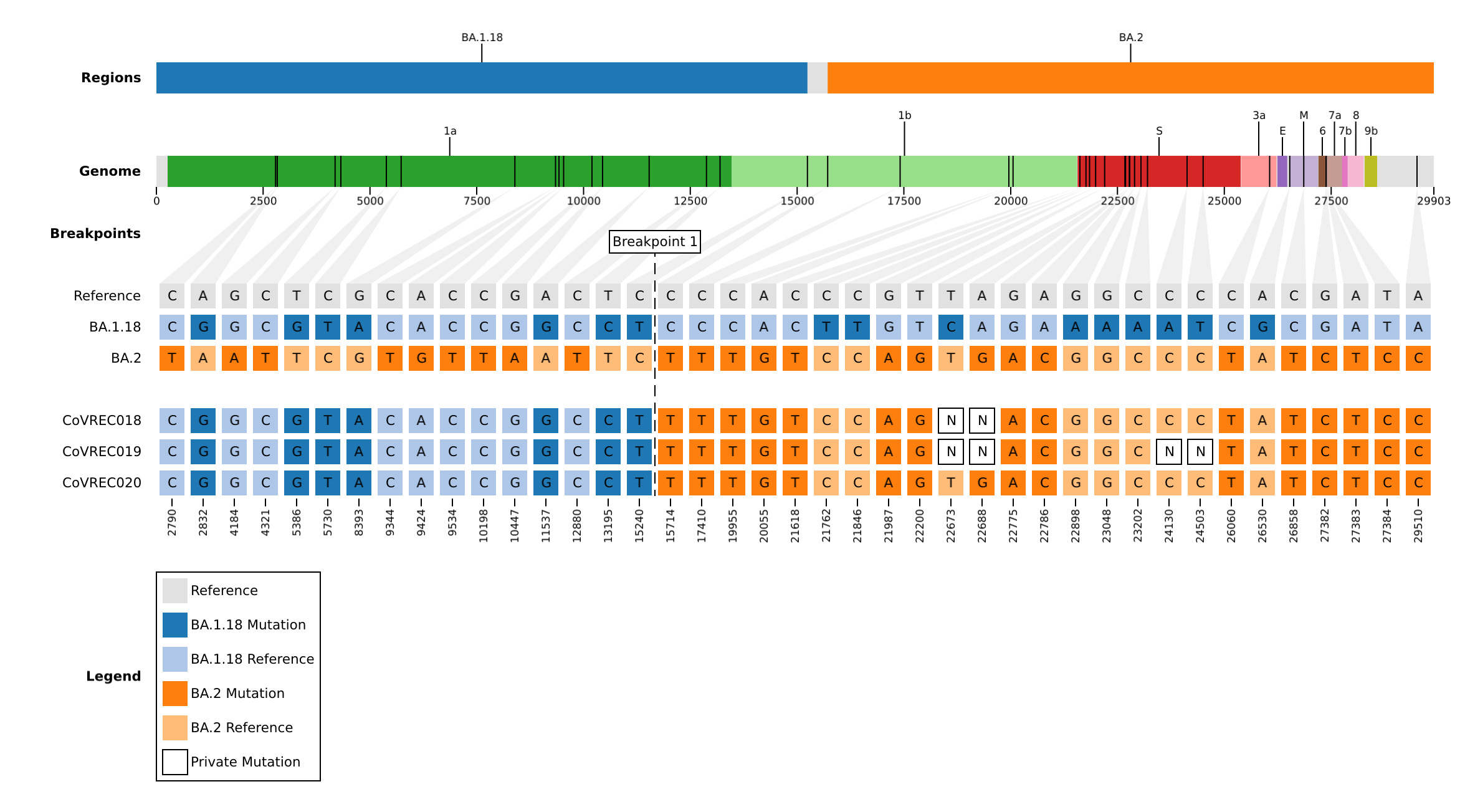


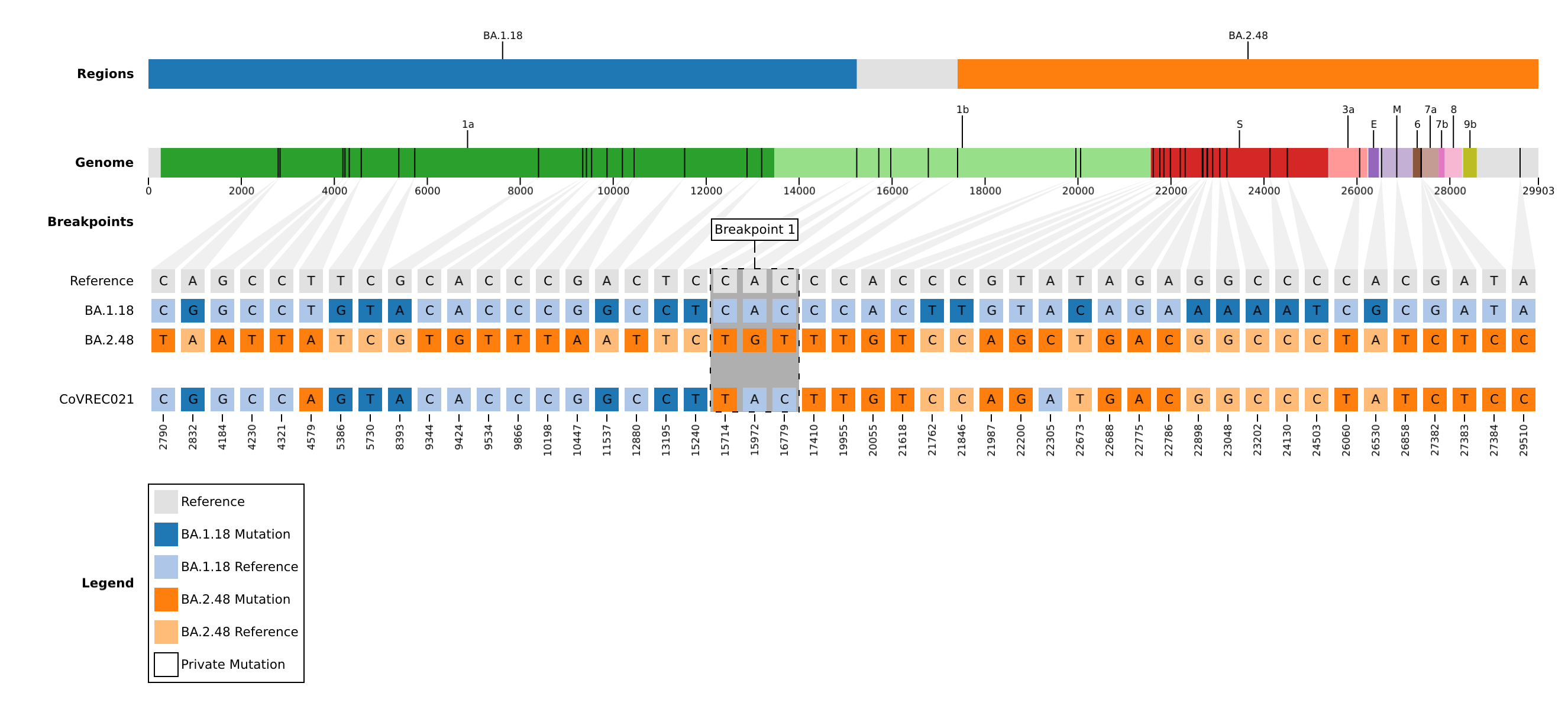


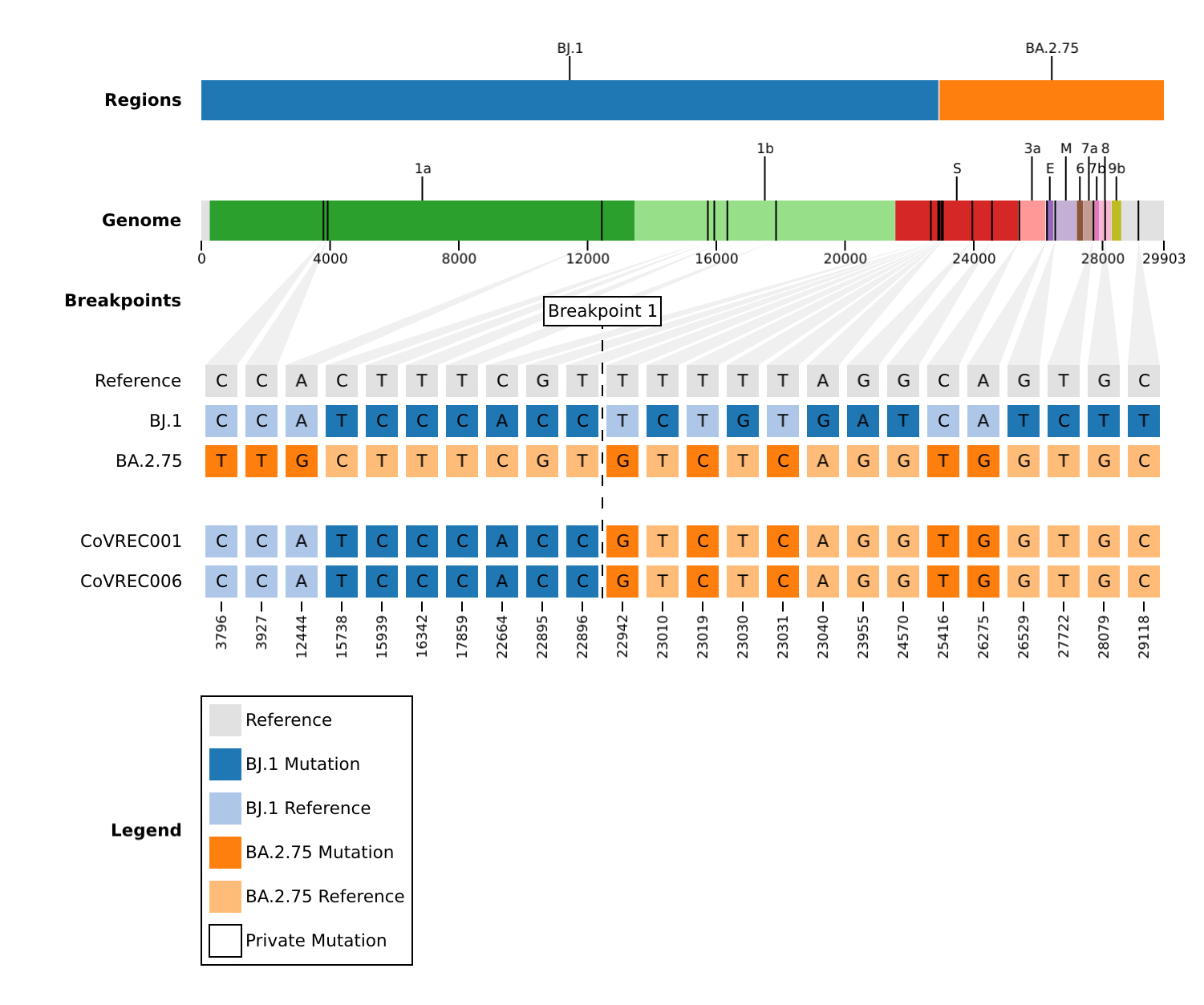
**Figure S2:** Representative mosaic structures showing of some of the putative SARS-CoV-2 recombinant sequences characterised in Botswana.


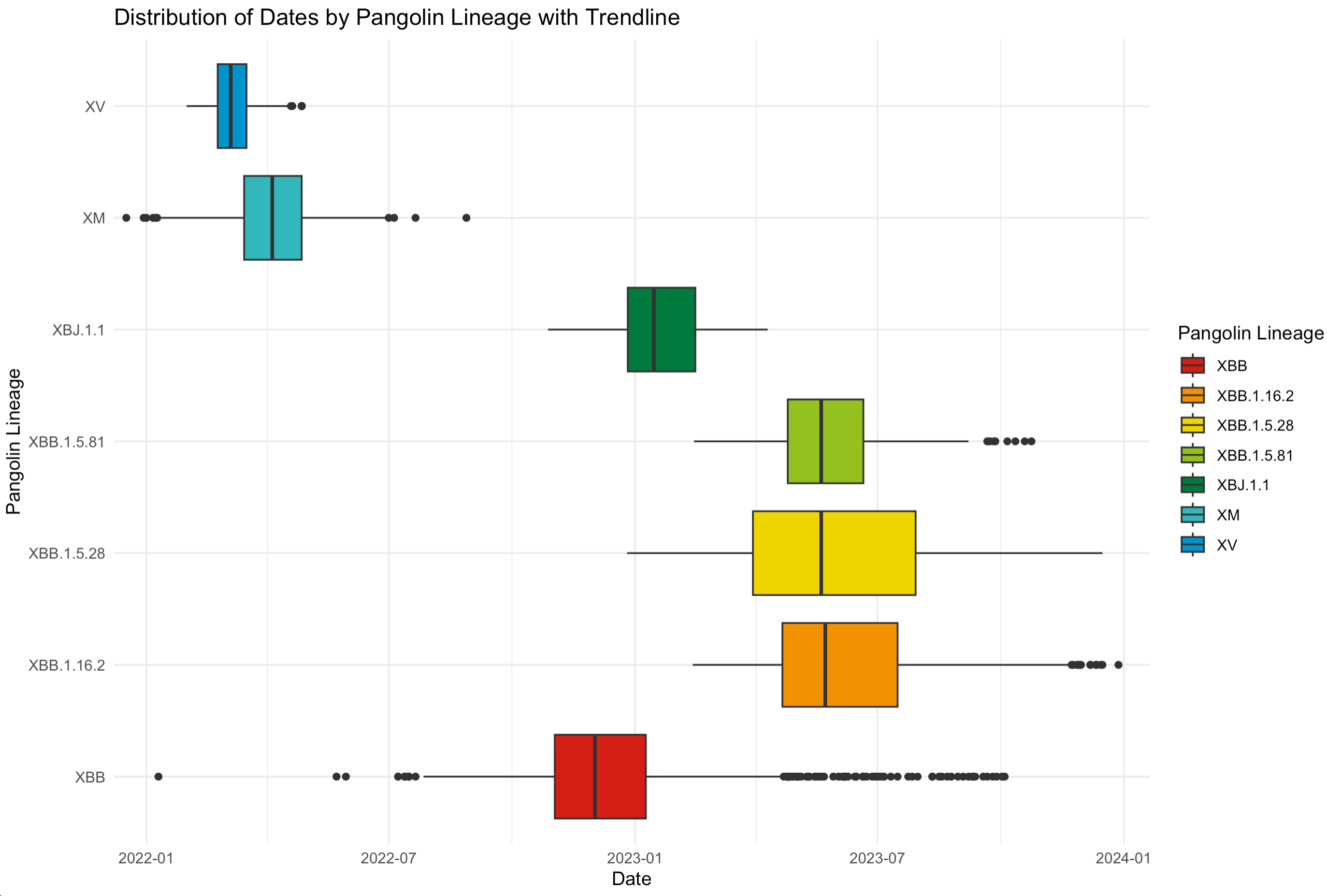


**Supplementary Figure 3A.** Boxplots showing the distribution of sampling dates for recombinant samples

.

***
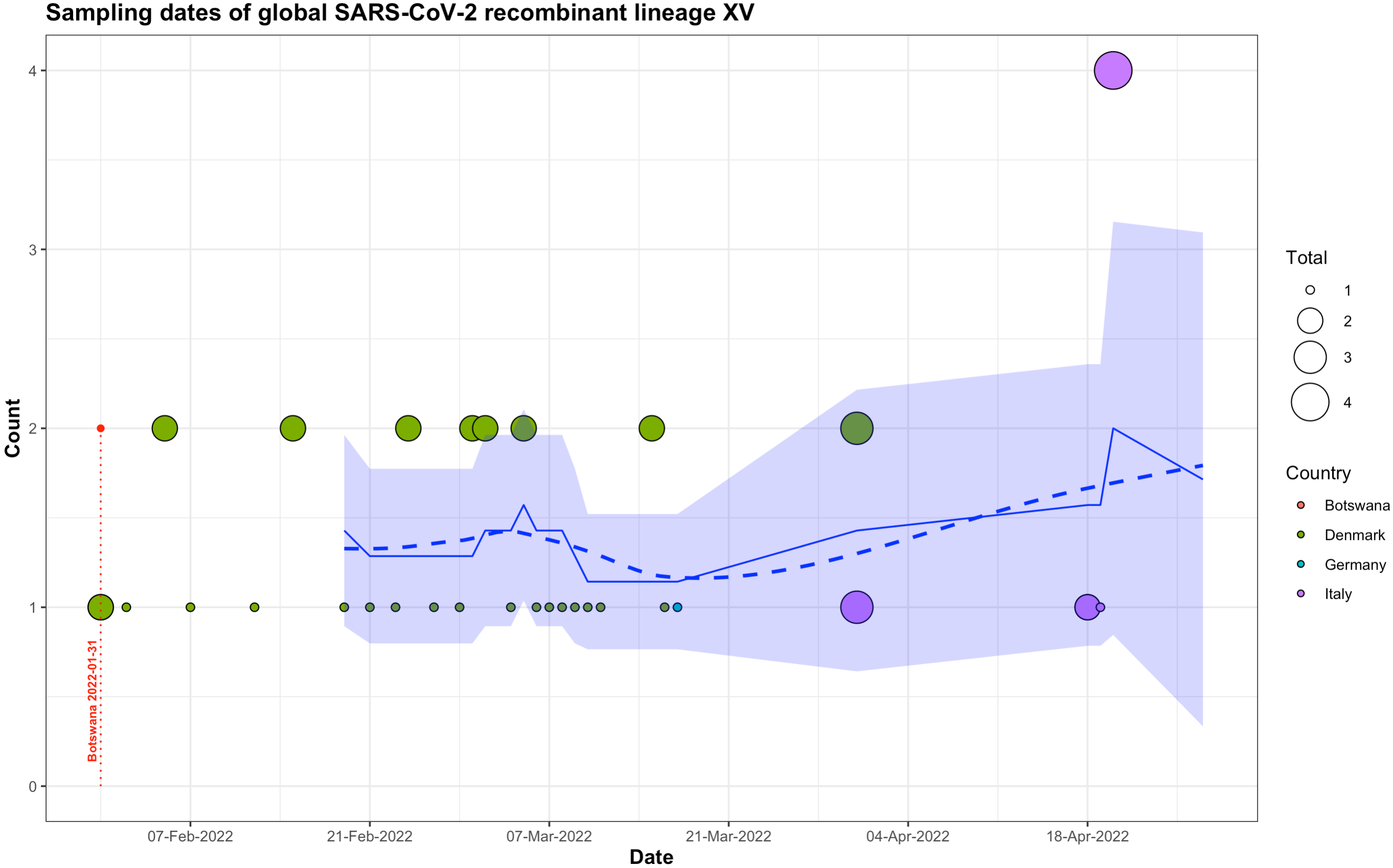

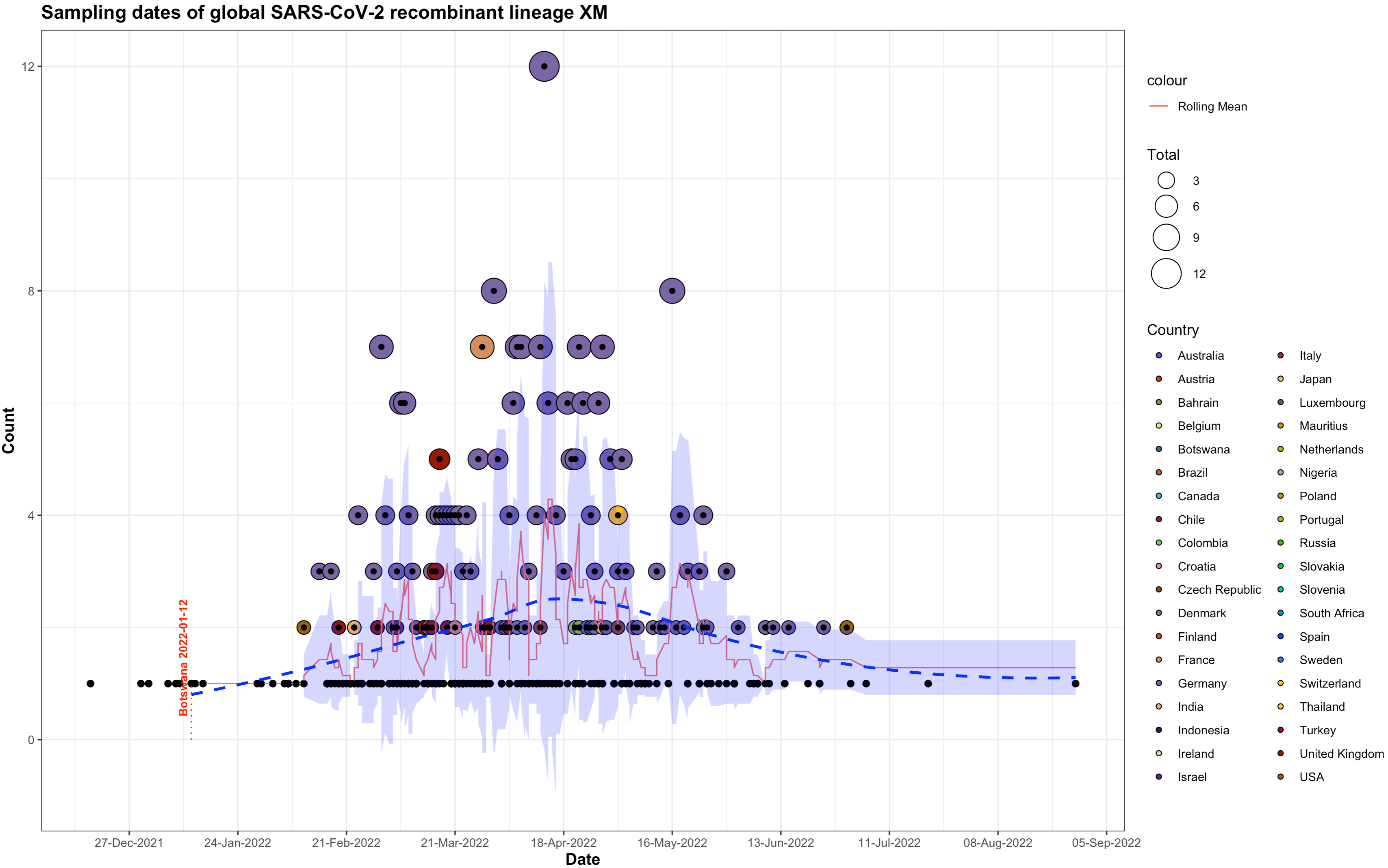
***

**
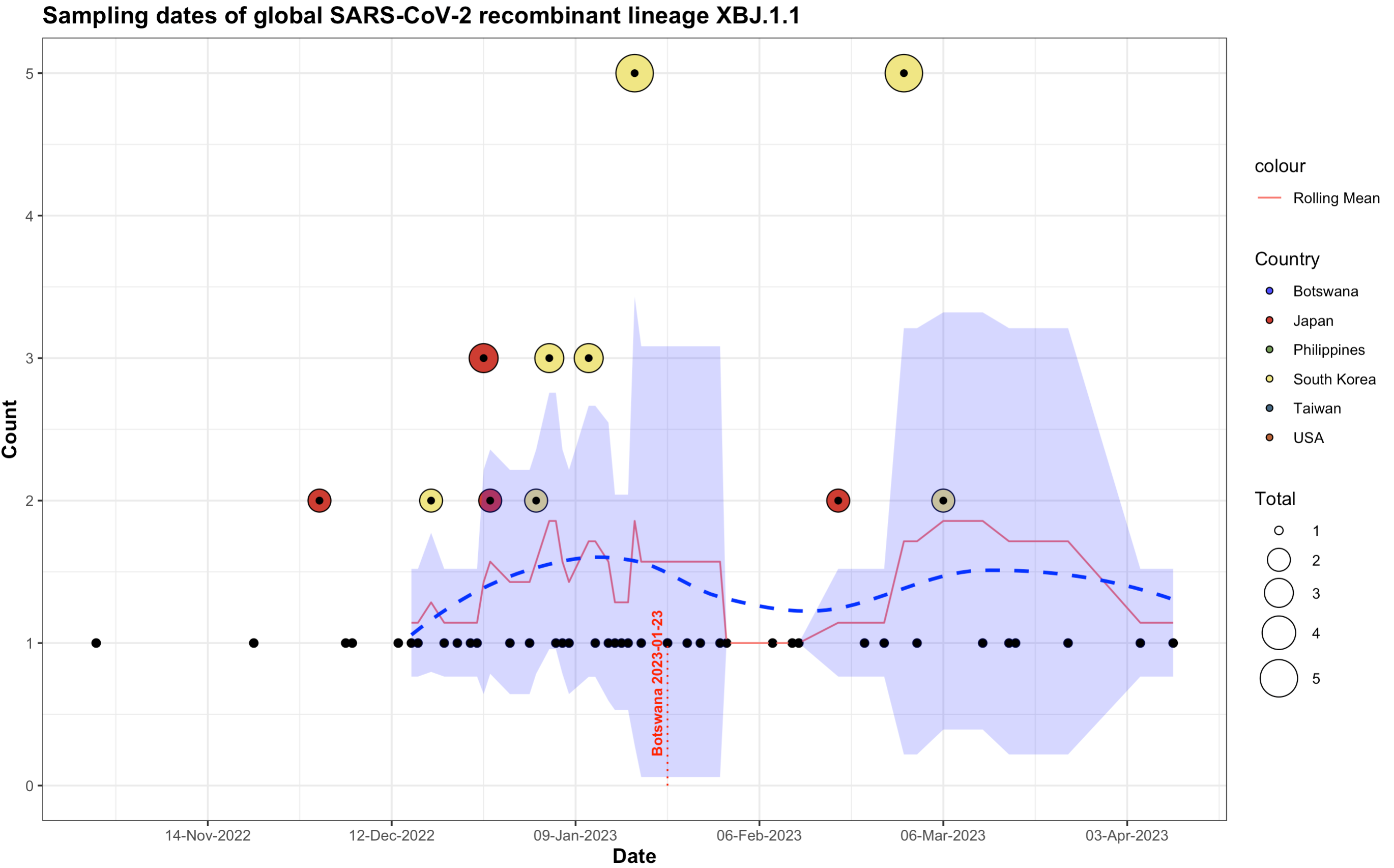

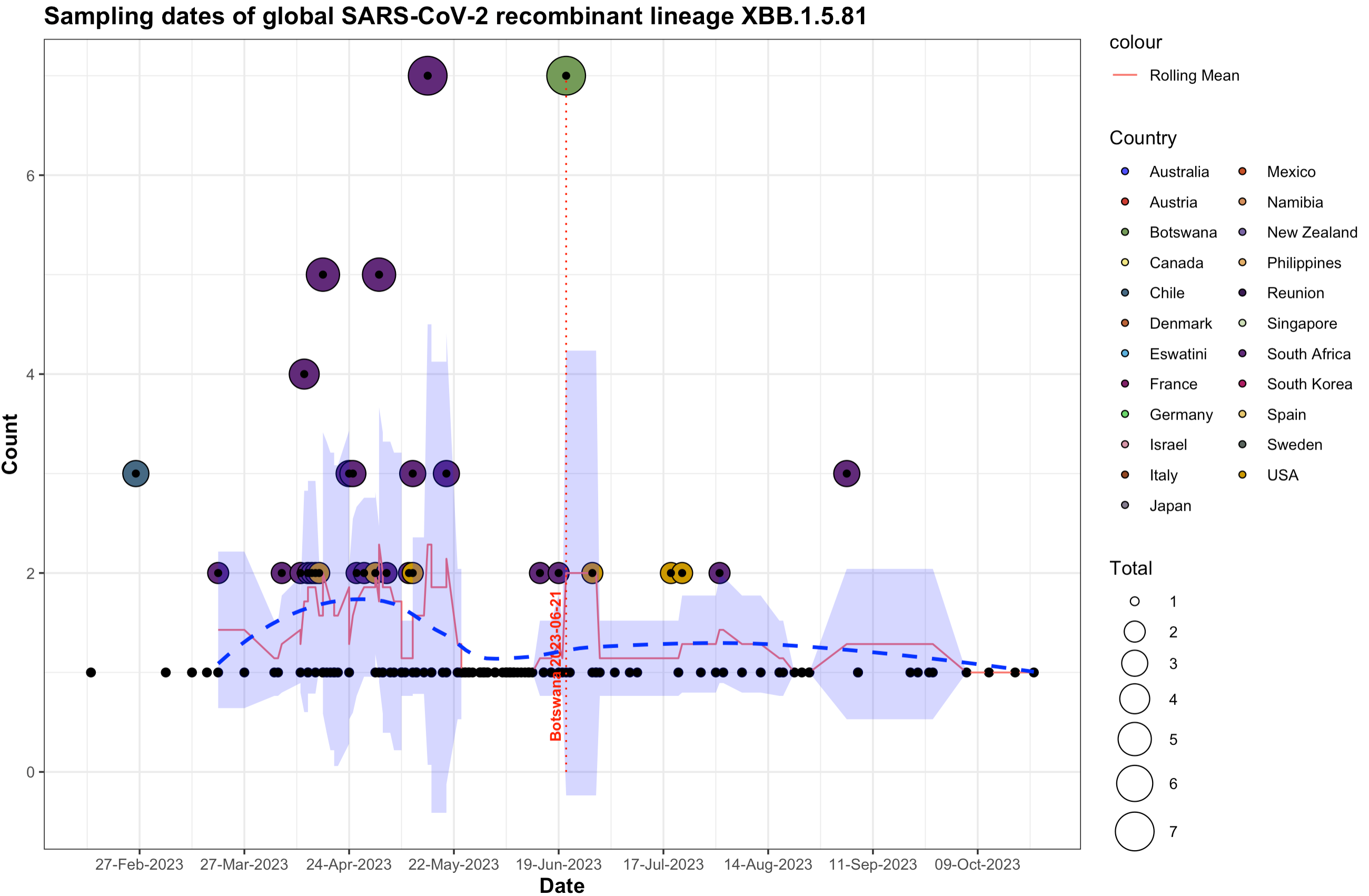
**

***
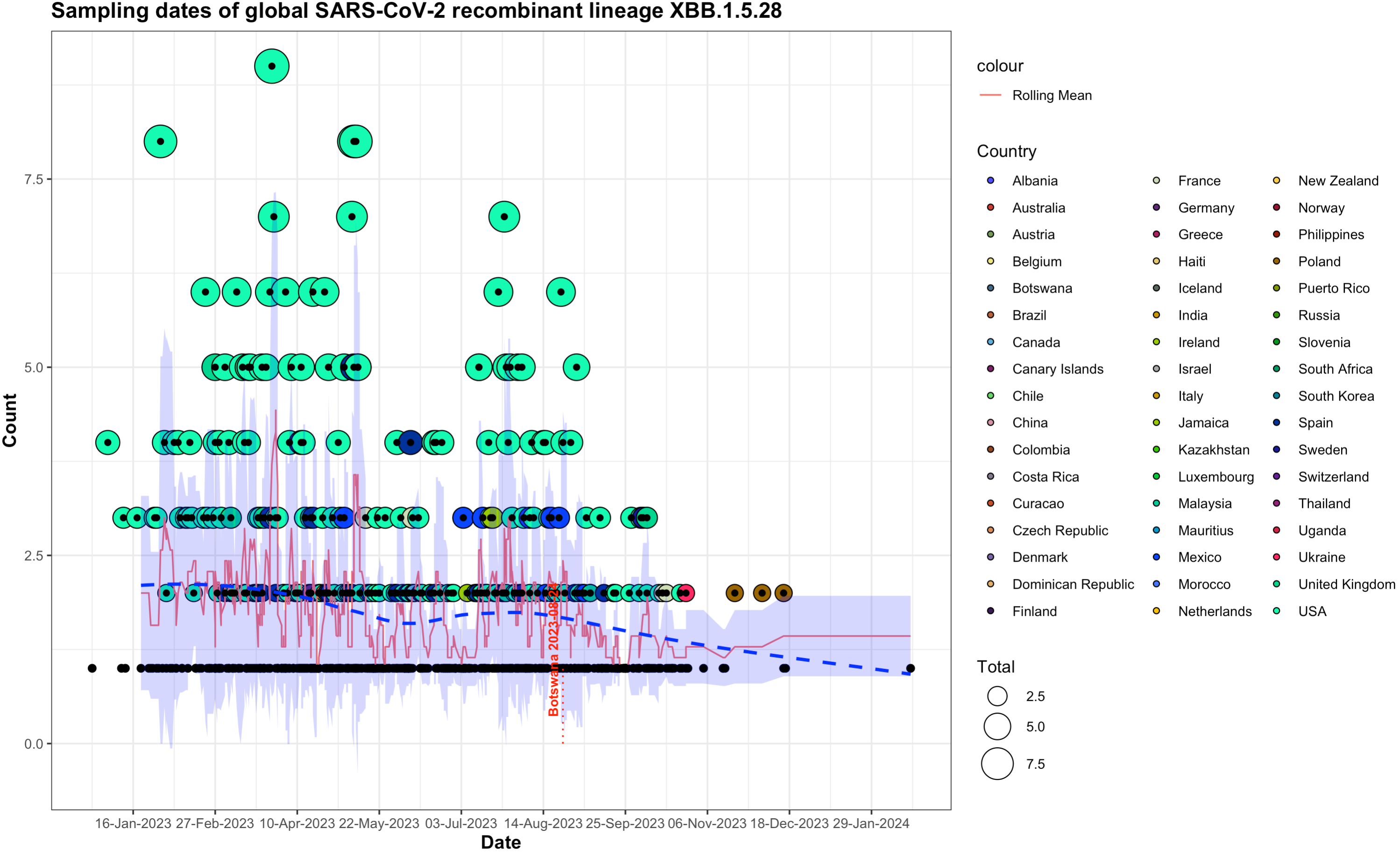
***

***
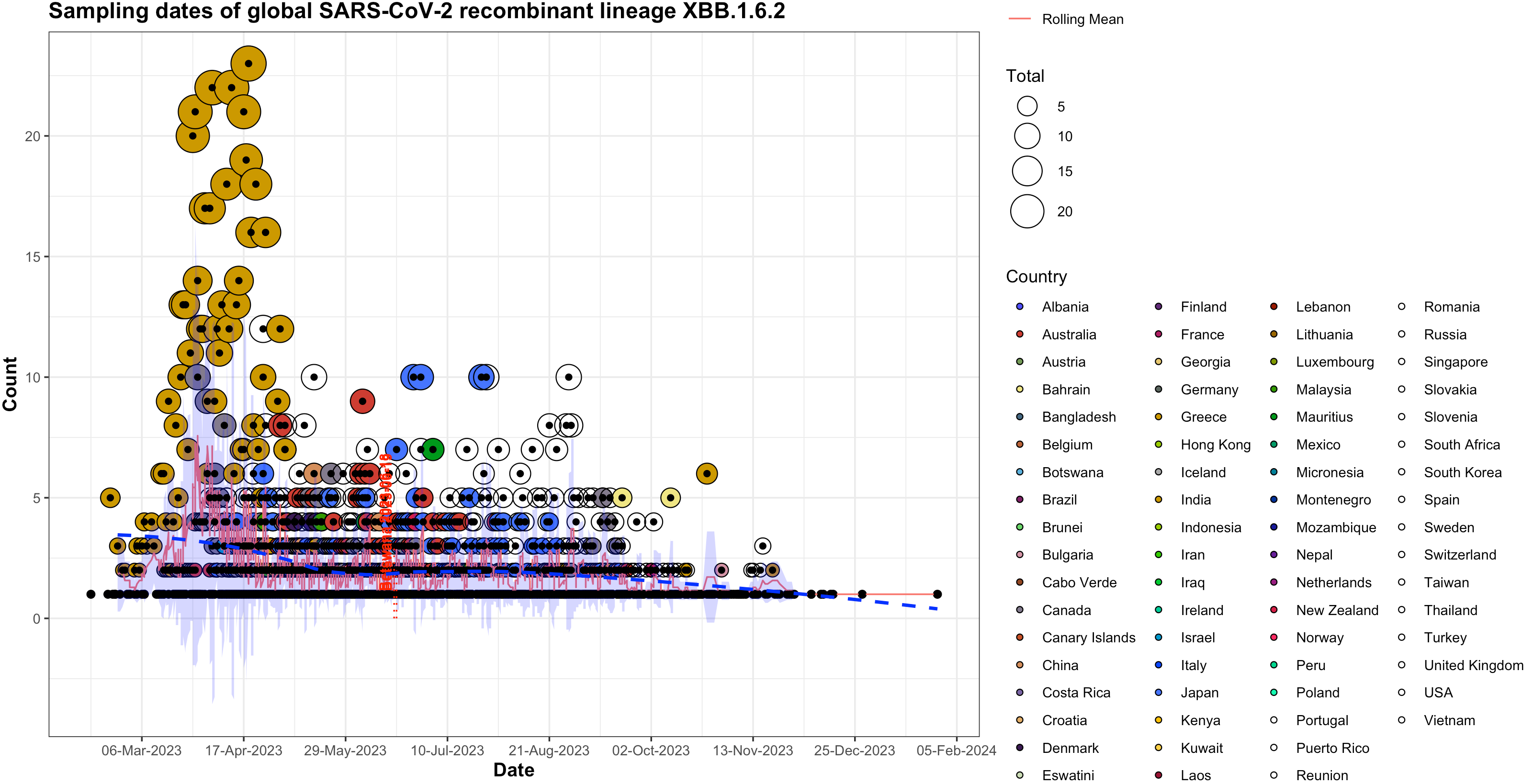
***

**Figure S3B.** Timelines of sampling dates of various recombinant lineages based on GISAID data. The sampling times of BW samples are indicated.

**A**


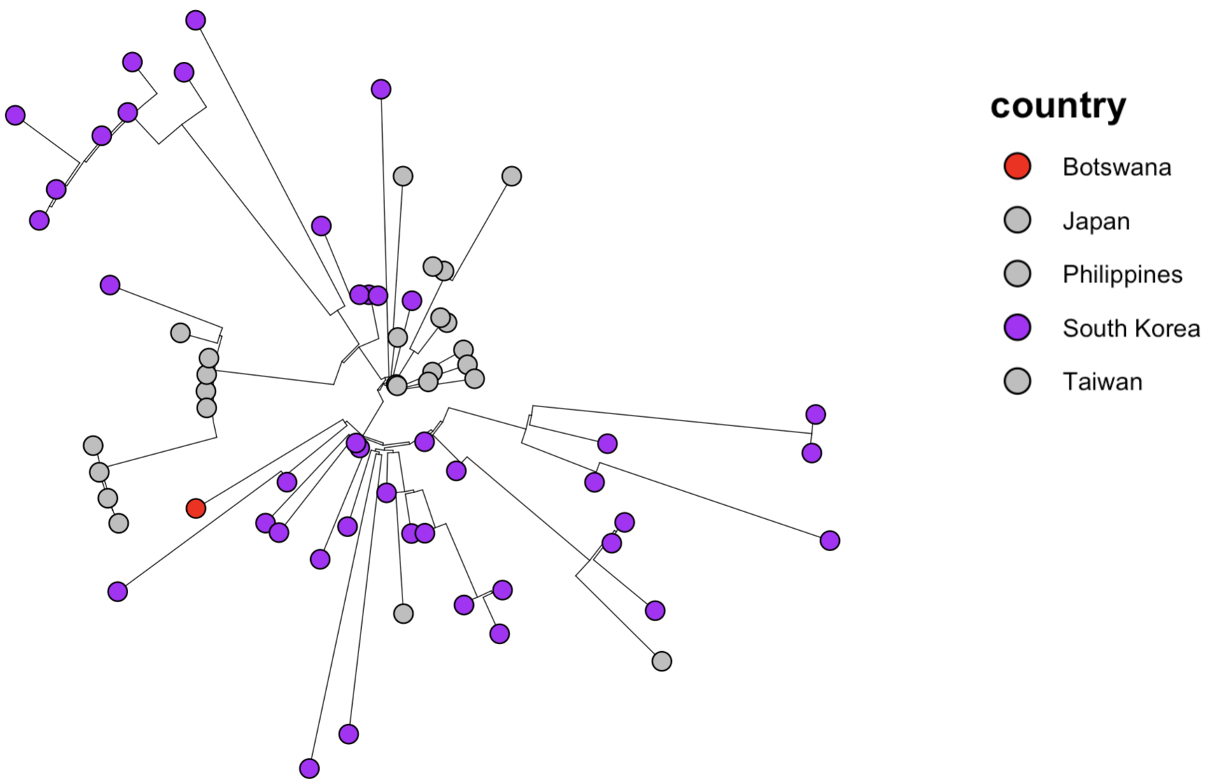


^
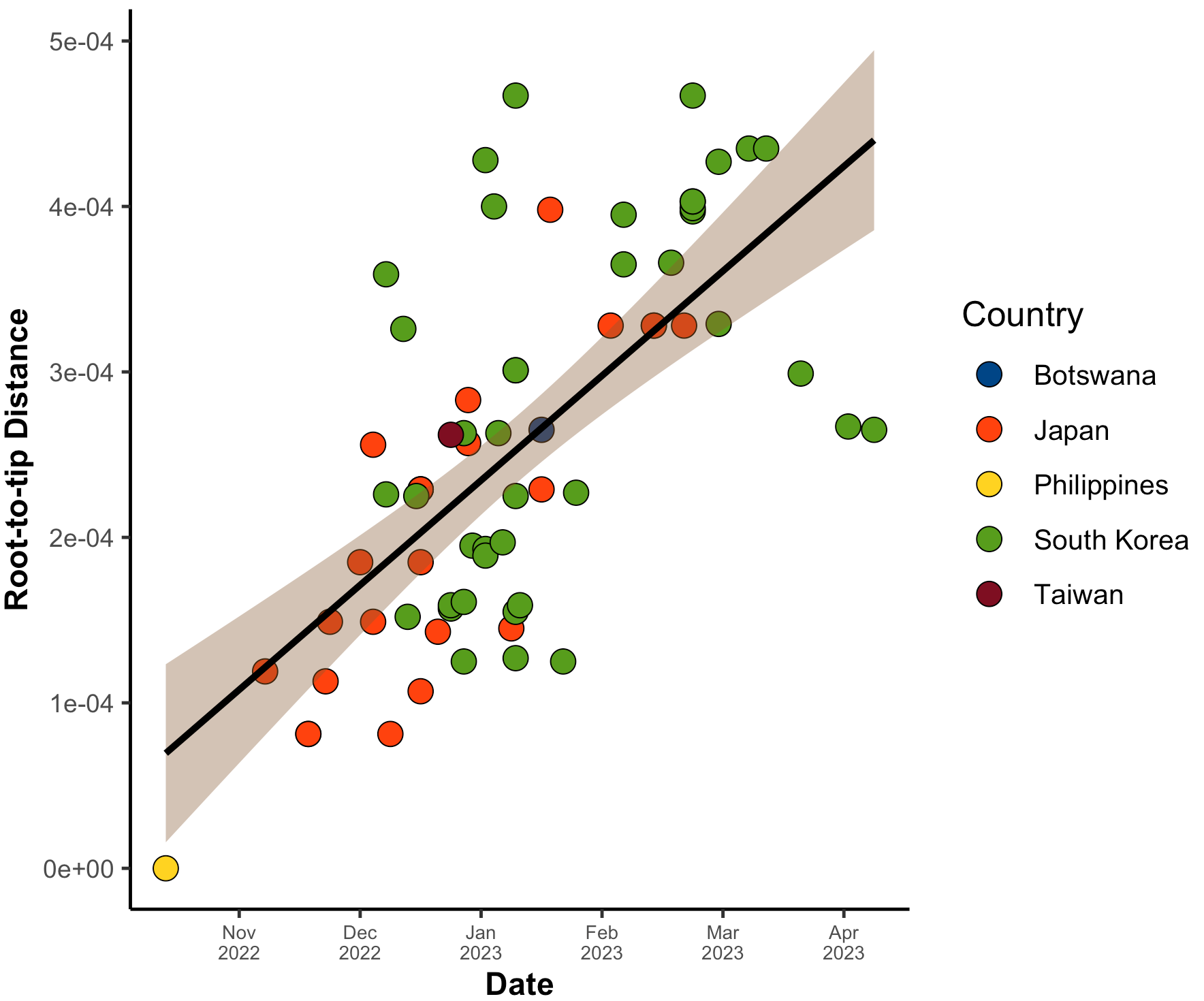
^

**B**


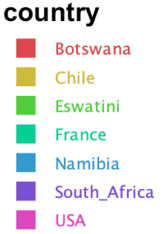

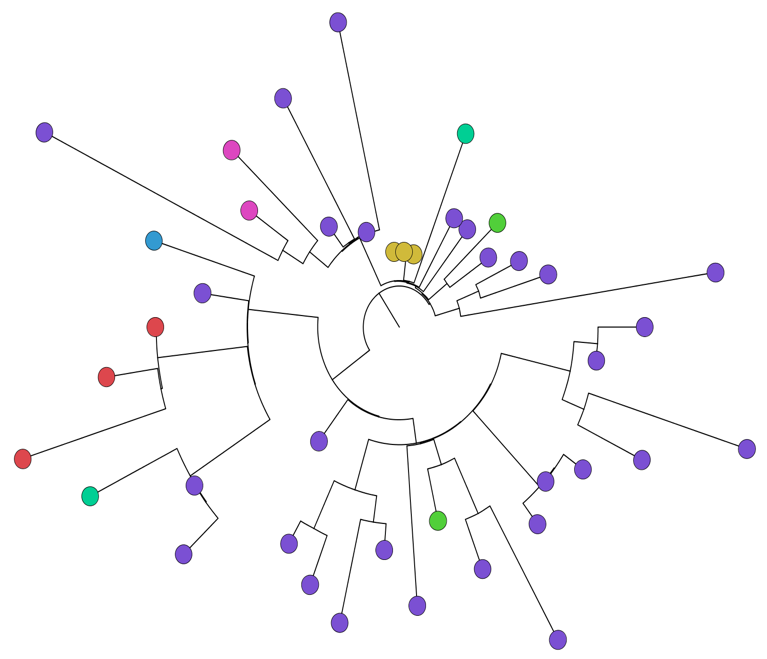


**C**

**E**


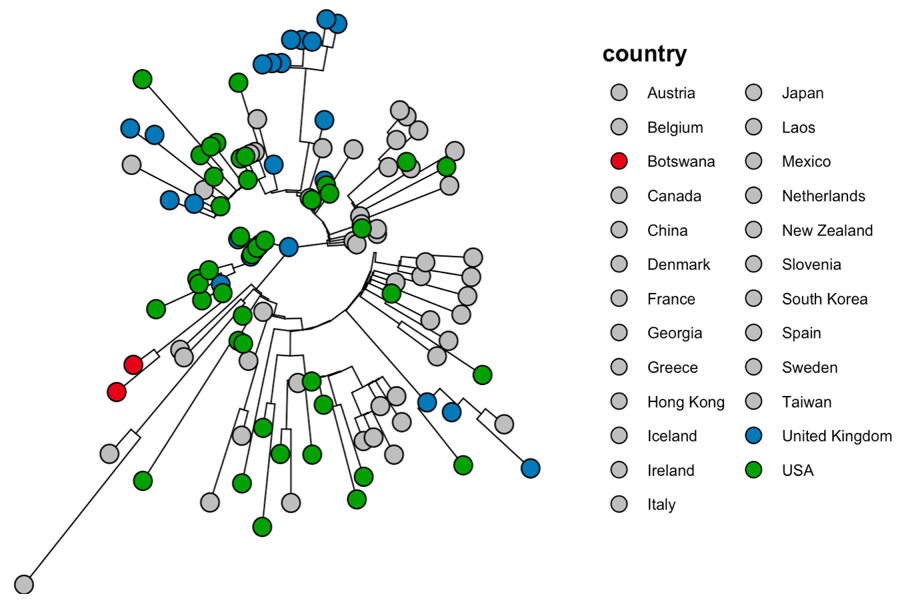


**Figure S4:** Maximum-likelihood phylogenetic trees showing the relatedness of: all recombinant sequences from Botswana and other representative reference sequences **A.** XBJ.1.1. Representative graphs of root-to-tip regression analysis showing correlation of sequence based on diversity and time. **B.** XBJ.1.1. ML phylogenetic trees were for **C.** XM., and **D.** XBB.1.5. annotated based on circular topology. Only sub-branches with posterior probability ≥ 0.90 are shown.


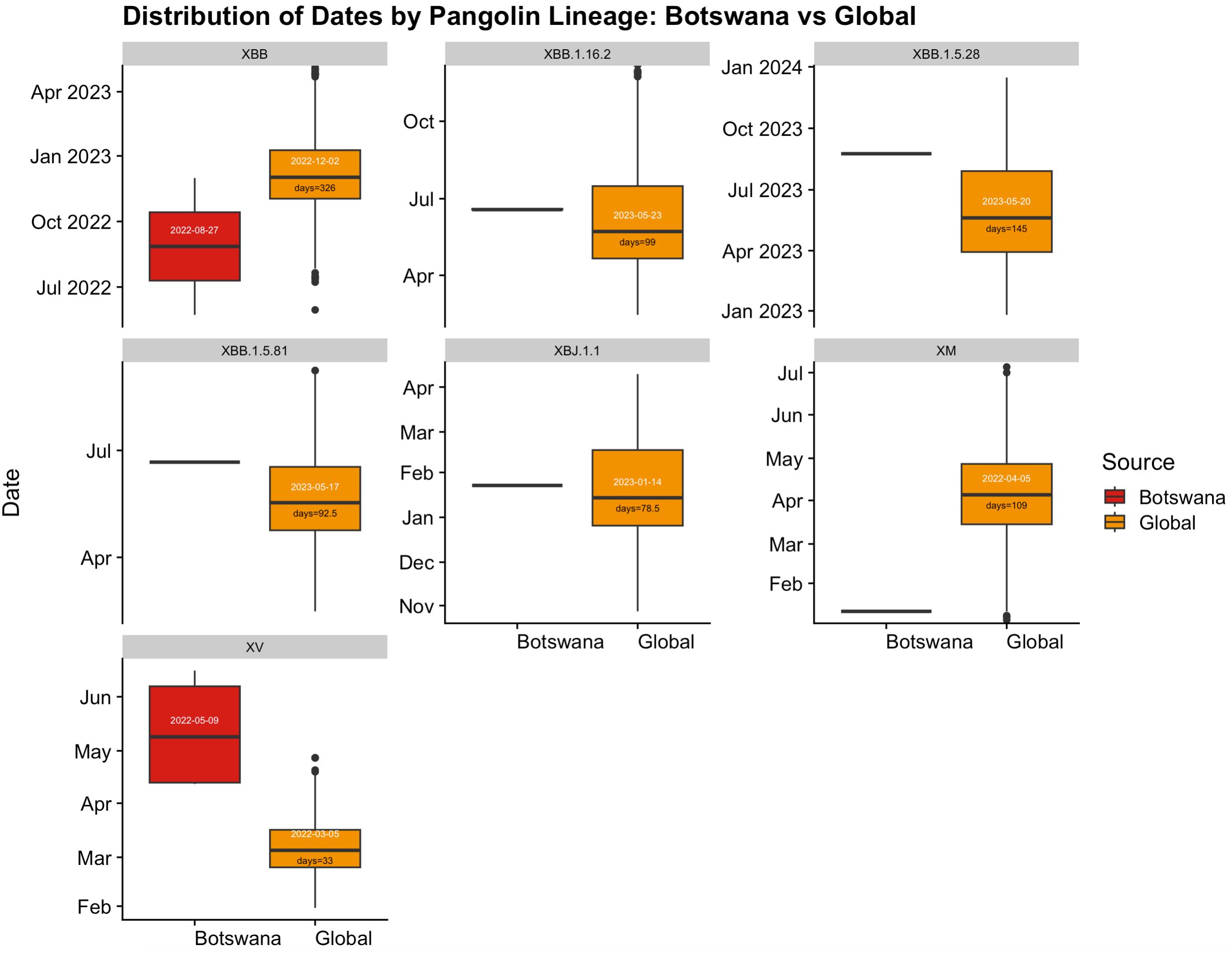


**Figure S5:** Comparison is shown between Botswana versus the Global sequences.
